# Structural transitions during large ribosomal subunit maturation analyzed by tethered nuclease structure probing in *S. cerevisiae*

**DOI:** 10.1371/journal.pone.0179405

**Published:** 2017-07-07

**Authors:** Gisela Pöll, Christian Müller, Malena Bodden, Fabian Teubl, Norbert Eichner, Gerhard Lehmann, Joachim Griesenbeck, Herbert Tschochner, Philipp Milkereit

**Affiliations:** 1 Lehrstuhl für Biochemie III, Universität Regensburg, Regensburg, Germany; 2 Lehrstuhl für Biochemie I, Universität Regensburg, Regensburg, Germany; University of Edinburgh, UNITED KINGDOM

## Abstract

Yeast large ribosomal subunit (LSU) precursors are subject to substantial changes in protein composition during their maturation due to coordinated transient interactions with a large number of ribosome biogenesis factors and due to the assembly of ribosomal proteins. These compositional changes go along with stepwise processing of LSU rRNA precursors and with specific rRNA folding events, as revealed by recent cryo-electron microscopy analyses of late nuclear and cytoplasmic LSU precursors. Here we aimed to analyze changes in the spatial rRNA surrounding of selected ribosomal proteins during yeast LSU maturation. For this we combined a recently developed tethered tertiary structure probing approach with both targeted and high throughput readout strategies. Several structural features of late LSU precursors were faithfully detected by this procedure. In addition, the obtained data let us suggest that early rRNA precursor processing events are accompanied by a global transition from a flexible to a spatially restricted rRNA conformation. For intermediate LSU precursors a number of structural hallmarks could be addressed which include the fold of the internal transcribed spacer between 5.8S rRNA and 25S rRNA, the orientation of the central protuberance and the spatial organization of the interface between LSU rRNA domains I and III.

## Introduction

Eukaryotic cytoplasmic ribosomes consist of 4 or more ribosomal RNAs (rRNA) and more than 70 proteins (r-protein). Their production requires the synthesis, assembly and folding of these components, extensive modification and processing of rRNA precursors (pre-rRNA), and transport of ribosomal precursor particles from the nucleolus, where most assembly steps take place, to the cytoplasm. These different aspects of eukaryotic ribosome maturation have been investigated intensively in the unicellular organism *S*. *cerevisiae* (hereafter called yeast) (Reviewed in [1,2]). In these studies more than 100 factors have been identified which transiently interact with ribosomal precursors during their maturation. Furthermore, yeast mutant analyses showed that association and release of specific biogenesis factors, r-protein assembly events and the processing and transport of pre-rRNA are functionally linked and coordinated with each other. As a likely consequence of this, and possibly the specific kinetics of different ribosomal maturation steps, various ribosomal precursor populations of largely defined composition and pre-rRNA processing state can be affinity purified from wildtype yeast cells using appropriately selected tagged ribosome biogenesis factors. In case of the large ribosomal subunit (LSU) several precursor populations can be distinguished by their protein composition and pre-rRNA processing state. Early nuclear LSU precursors, (hereafter termed 27SA-preLSUs) contain 27SA pre-rRNA which still includes spacer sequences 5’ of 5.8S rRNA (internal transcribed spacer 1 = ITS1) and between 25S rRNA and 5.8S rRNA (internal transcribed spacer 2 = ITS2, see S1 Fig for an overview). They can be enriched by affinity purification of several biogenesis factors, among them Noc1 [3]. Intermediate nuclear LSU precursors can be affinity purified via tagged Noc3 and other biogenesis factors and they contain 27SB pre-rRNA in which the 5’ flanking ITS1 spacer sequences were removed (hereafter called 27SB-preLSUs, see S1 Fig and S2B Fig) [4–7]. Endonucleolytic cleavage in the ITS2 at site C2 between 5.8S rRNA and 25S rRNA and initial trimming of spacer regions leads to 7S pre-rRNA containing nuclear particles (hereafter called 7S-preLSUs, see S1 Fig) which associate again with a specific set of ribosome biogenesis factors. A few biogenesis factors, among them Lsg1 [8,9], specifically bind to late cytoplasmic LSU precursor particles and mature free LSUs which contain fully trimmed 25S rRNAs (hereafter called 25S-preLSUs, see S1 Fig).

**Fig 1:**
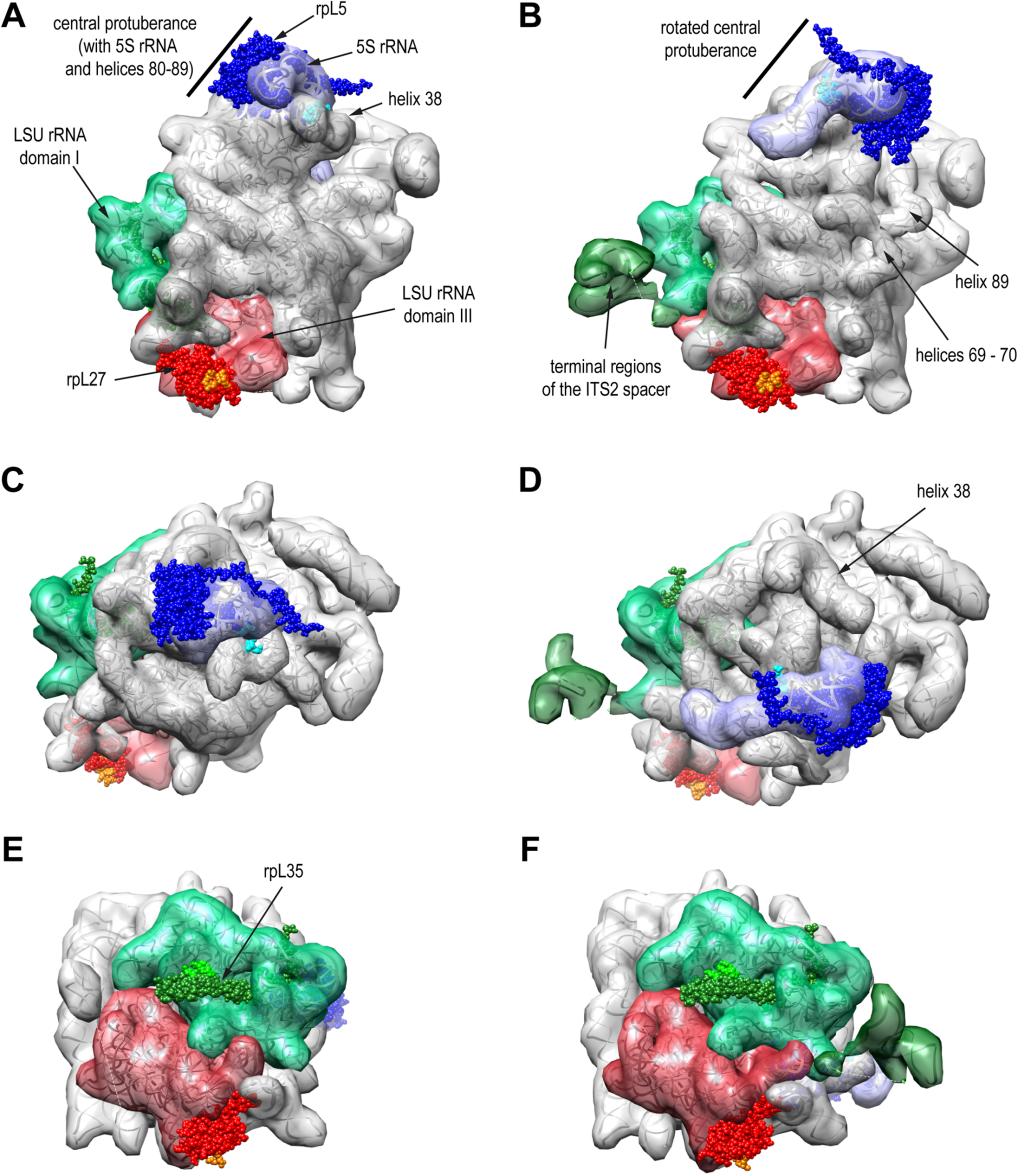
7S-preLSU and 25S-preLSU rRNA tertiary structure with binding sites of ribosomal proteins used for tethered structure probing. Structures of rRNA, rpL5, rpL27 and rpL35 in 25S-preLSUs (A, C, D) or 7S-preLSUS (B, D, F) are shown in three orientations with either the subunit interface side (A and B), the central protuberance (C and D) or the region formed by LSU rRNA domains I and III (E and F) in the center. In (A-F) the transparent surface of RNA filtered to 20 Angstrom resolution is superimposed on the RNA backbone. The surface representation of 5S rRNA is in blue, of LSU rRNA domain I and terminal ITS2 spacer regions in green and dark green, of LSU rRNA domain III in red and of other rRNA in grey. Spherical atomic models of rpL5, rpL27 and rpL35 are visualized in blue, red and green with the amino-terminal regions in light blue, orange and light green. Pdb files 5apo and 3jct used to create this figure were published in [12,16].

Structures of several 7S-preLSU and late 25S-preLSU populations could be resolved at intermediate to near atomic resolution by single molecule cryo-electron microscopy thereby allowing comparison with structures of yeast mature ribosomes in various conformational states solved either by X-ray crystallography or cryo-electron microscopy (reviewed in [10,11]). Accordingly, the rRNA fold observed in late 25S-preLSUs closely resembles the one in mature ribosomes [12–15]. In 7S-preLSUs the rRNA fold of mature ribosomes is already largely established with only a few pre-ribosomal regions showing substantial differences [16]. That is reorientation and partial unfolding of some helices in the ribosomal subunit interface region (LSU rRNA helices 69–71) and a complete reorientation of the 5S rRNA and the adjacent helices 80–87, 89 and 38 which form all together a prominent substructure in the LSU appearing as a central protuberance (see Fig 1 for an overview). During the maturation of 7S-preLSUs to 25S-preLSUs areas with a pre-mature rRNA fold are hotspots for dynamic changes in factor and r-protein composition. Thus, a large number of biogenesis factors (including Rea1, Rix1, Sda1, Nmd3, Rsa4, Nog2, Nog1, Rrs1, Rpf2, Nsa2 and Cgr1) transiently interact on the way from 7S-preLSUs to 25S-preLSUs with these pre-rRNA sites. In addition four out of the six r-proteins with late assembly characteristics are stably incorporated into LSU-precursors during these maturation steps at these sites and stabilize the respective rRNA fold of mature ribosomes [17,18]. These four r-proteins are designated rpL10, rpL29, rpL40 and rpL42 according to the *S*. *cerevisiae* standard nomenclature (www.yeastgenome.org, [19]) and they are named uL16, eL29, eL40 and eL42 according to the universal nomenclature introduced by Ban *et al*. [20]. A second major group of factors, including Nop53, Nop7, Rlp7, Cic1 and Nop15, binds at and around the spacer sequences between 5.8S and 25S rRNA in a foot-like structure. Spacer trimming goes along with the dissociation of these factors from LSU precursors.

We recently developed an approach to analyse the local rRNA surrounding of ribosomal proteins [21]. Selected ribosomal proteins were expressed in yeast cells in fusion with the calcium dependent micrococcal nuclease (MNase) to tether the enzyme to specific ribosomal sites. After preparation of cellular extracts the MNase was activated and resulting cleavages in mature rRNA were mapped by primer extension analyses. Dominant cuts were found to be restricted to accessible single stranded rRNA regions at the respective neighbouring ribosomal surface. In the present work we aimed to further improve this approach by using high throughput sequencing methodology as readout to facilitate the screening and quantitation of fusion protein dependent cuts in large or heterogeneous ribonucleoprotein complexes. We chose to test the performance of this approach using affinity purified late cytoplasmic 25S-preLSUs for which near atomic resolution structure data exist. Furthermore, we aimed to analyse the spatial organisation of early 27SA-preLSUs and 27SB-preLSUs. We decided to probe the structure of these LSU precursors with a MNase fusion of the central protuberance binding r-protein rpL5 (named uL18 in the universal nomenclature [20]) to test the orientation and tertiary rRNA fold of the protuberance and its environment (see Fig 1 for an overview). In addition we performed a tertiary structure analysis of the pre-ribosomal region around the interface of LSU rRNA secondary structure domains III and I, including the 5.8S rRNA and the ITS2 spacer region between 5.8S rRNA and 25S rRNA. Previous experiments indicated that secondary structure formation of this rRNA region critically depends on a number of r-proteins and biogenesis factors binding close by [22–28]. In addition, alternative secondary structure models have been proposed for the central part of the ITS2 spacer [29–31] and it has been suggested that productive processing of the spacer might require its remodelling during LSU maturation [25,28,32,33]. We chose to probe structural rearrangements in this region in the local rRNA surrounding of the two r-proteins rpL27 and rpL35 (named eL27 and uL29 in the universal nomenclature [20]). RpL35 directly binds to the 5.8S rRNA next to the ribosomal polypeptide exit tunnel and rpL27 binds to LSU rRNA structure domain III close to the 5.8S rRNA 3’ end and the ribosomal subunit interface (see Fig 1 for an overview).

## Results and discussion

### Evidence for global structural reorganization of LSU precursors during maturation of 27SA-preLSUs to 27SB-preLSUs

**Fig 2:**
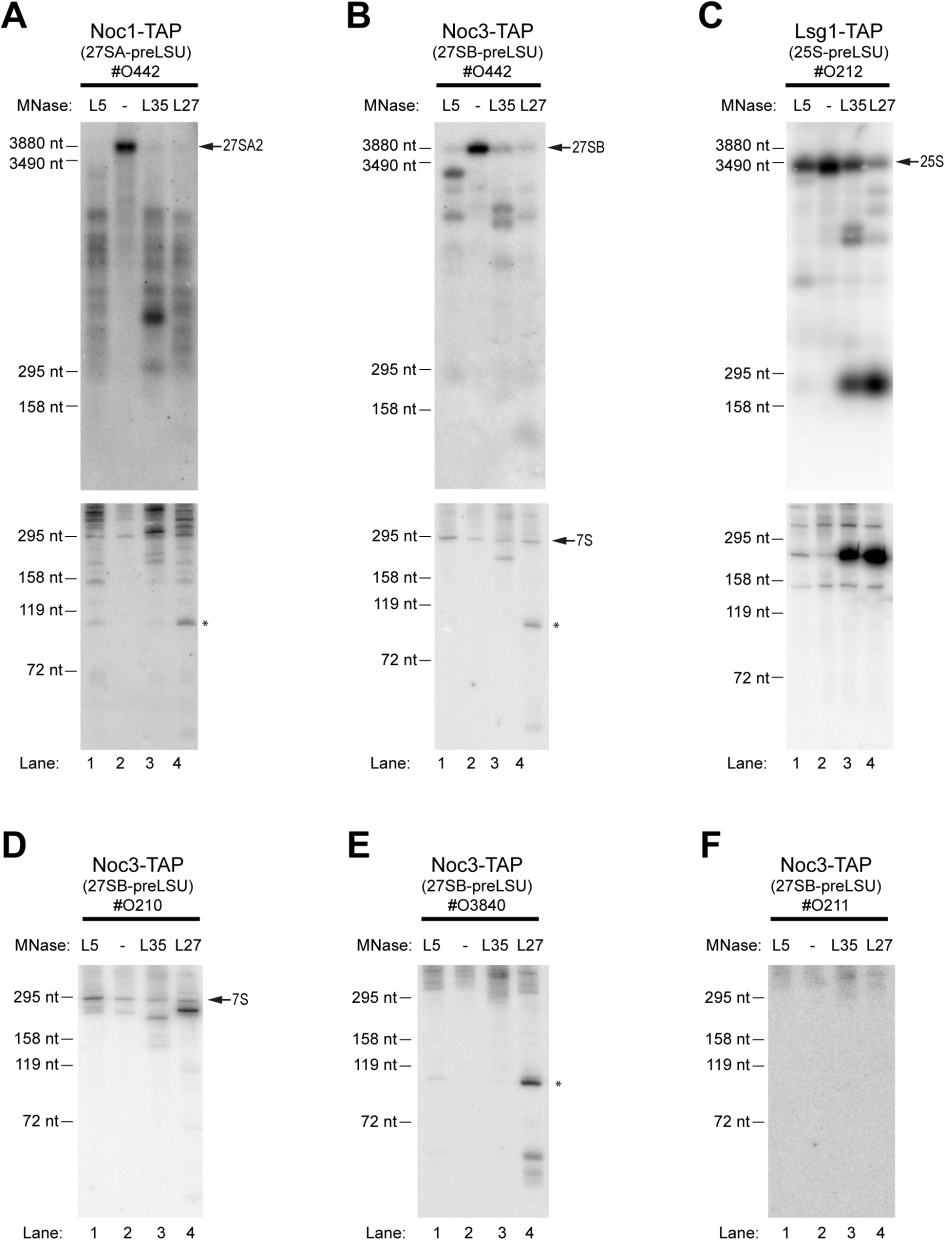
Tethered MNase cleavages in 27SA-preLSUs, 27SB-preLSUS and 25S-preLSUS as analyzed by northern blotting. 27SA-preLSUs (A), 27SB-preLSUS (B, D-F) and 25S-preLSUS (C) were affinity purified using the indicated TAP tagged biogenesis factors from yeast strains expressing no MNase (lane 2) or MNase in fusion with rpL5 (lane 1), rpL35 (lane 3) or rpL27 (lane 4). After activation of MNase by addition of calcium for 10 minutes at 16°C the resulting rRNA fragments were analyzed by Northern Blotting using the indicated probes (# as prefix, see S1 Fig for rRNA regions recognized by the respective probes). In the upper panel in (A—C) RNA was separated by size by agarose gel electrophoresis, in (D-F) and in the lower panels in (A-C) by acrylamide gel electrophoresis. A ~110 nucleotide long fragment covering the central ITS2 region detected by probes #442 (B) and #3840 (E) is highlighted by an asterisk. Running behavior of RNA fragments of defined length are indicated on the left.

To analyze the local rRNA surrounding of rpL5, rpL27 and rpL35 we made use of three yeast strains solely expressing the respective proteins in fusion with MNase (see Materials and Methods). Each of the three strains was further genetically modified to express chromosomally encoded tandem affinity purification (TAP) tagged versions of either Noc1, Noc3 or Lsg1. As a control, strains were generated which express TAP tagged Noc1, Noc3 or Lsg1 but no MNase fusion potein. Growth characteristics in glucose containing medium of the resulting twelve yeast strains were analyzed by measurement of optical densities (see Materials and Methods). They indicated only slight effects of MNase (< 25% increase in doubling times) and TAP fusions (< 25% increase in doubling time for Lsg1-TAP) on the essential functions of the tagged proteins (see S3 Table). To probe the structure of the selected LSU precursors cellular lysates were prepared from all twelve yeast strains and the respective TAP tagged factors together with associated ribosomal particles were affinity purified (see Materials and Methods). Northern blotting analyses of rRNA precursors contained in the cellular lysates and the affinity purified fractions confirmed the efficient purification of 27SA-preLSUs, 27SB-preLSUs and 25S-preLSUs via Noc1-TAP, Noc3-TAP and Lsg1-TAP, respectively (S2 Fig). The nuclease activity of the different MNase–r-protein fusions in the affinity purified LSU precursors was stimulated by incubation for 10 minutes at 16°C in the presence of calcium. The resulting cleavages in rRNA precursors were first analyzed by northern blotting (Fig 2). The fastest and most drastic degradation of rRNA precursors was clearly observed in the 27SA-preLSUs associated with Noc1p. 27SA pre-rRNA contained in these LSU precursors was nearly fully converted into numerous fragments of heterogeneous size after the 10 minutes nuclease activation for each of the three MNase fusion proteins (compare levels of 27SA pre-rRNA in 27SA pre-LSUs containing no MNase fusion protein in Fig 2A, lane 2, with levels in 27SA-preLSUs containing MNase fused to rpL5 in lane 1, rpL35 in lane 3, or rpL27 in lane 4). Pre-rRNA degradation in 27SB-preLSUs purified via Noc3-TAP was less complete when applying the same conditions (Fig 2B, compare levels of 27SB pre-rRNA in lane 2 and lanes 1, 3 and 4) and it was the least advanced in Lsg1 associated 25S-preLSUs (Fig 2C, compare levels of 25S rRNA in lane 2 and lanes 1, 3 and 4). The pattern of cleavages generated by MNase fusions of rpL5, rpL27 and rpL35 appeared to be most similar in case of 27SA-preLSUs. However, subtle differences in the pre-rRNA fragmentation pattern of 27SA-preLSUs purified from strains expressing MNase in fusion with rpL5, rpL27 or rpL35 still argued for site specific integration of these proteins into early LSU precursors (Fig 2A, compare pattern of fragments in the lower panel). In case of 27SB-preLSUs and 25S-preLSUs much less rRNA fragments were detected and the fragment pattern observed was rather unique for each of the MNase fusion proteins (Fig 2B and 2C, compare fragment pattern in lanes 1, 3 and 4). Hence, 27SA-preLSUs were far more accessible for cleavages by MNase tethered to various ribosomal locations when compared to further matured 27SB-preLSUs or 25S-preLSUs. We take this as evidence for a rather flexible spatial organization of 27SA-preLSUs which is converted at the level of 27SB-preLSUs into a much more rigid and defined fold. Such major structural transition during maturation from 27SA-preLSUs to 27SB-preLSUs is consistent with results of previous biochemical experiments indicating a comparably weak association of most LSU r-proteins (including rpL5, rpL27 and rpL35) with 27SA-preLSUs [17].

### Random primer extension reactions combined with high-throughput sequencing as read out for tethered MNase assays

We next aimed to map the pre-rRNA 5’ ends generated by the MNase fusion proteins to deduce information on the local rRNA surrounding of these proteins in the purified LSU precursor populations. We focused on 27SB-preLSUs and 25S-preLSUs because of low 80S ribosomal contamination and the rather defined pre-rRNA fragmentation pattern observed in these cases. RNA was extracted from the respective calcium treated affinity purified fractions and primer extension reactions were performed using a reverse transcriptase (RT). In an attempt to screen for major cutting sites the primer extension reactions were performed with random primers and the 5’ and 3’ ends of the resulting cDNA fragments were determined by paired end high throughput sequencing (see Materials and Methods). The resulting data were further evaluated by statistical methods developed by Kielpinsky and colleagues [34]. This allowed for prediction of the percentage of all cDNA fragments spanning a LSU pre-rRNA region for which the reverse transcription reaction was terminated at a given position in this region (Termination to Coverage Ratio at each nucleotide position, hereafter abbreviated as TCR). When analyzing the coverage of cDNA fragments in LSU pre-rRNA we realized that the random priming of reverse transcription reactions was not evenly distributed. To obtain an estimate of a possibly resulting uneven distribution of random stops in the primer extension reactions we created for each dataset 100 simulations in which the observed priming sites were combined with a randomly chosen fragment length from the pool of observed cDNA fragment lengths. The local noise of primer extension stops in each rRNA region was then estimated using the maximum TCR value in this region in all simulated datasets (see Materials and Methods for details).

**Fig 3:**
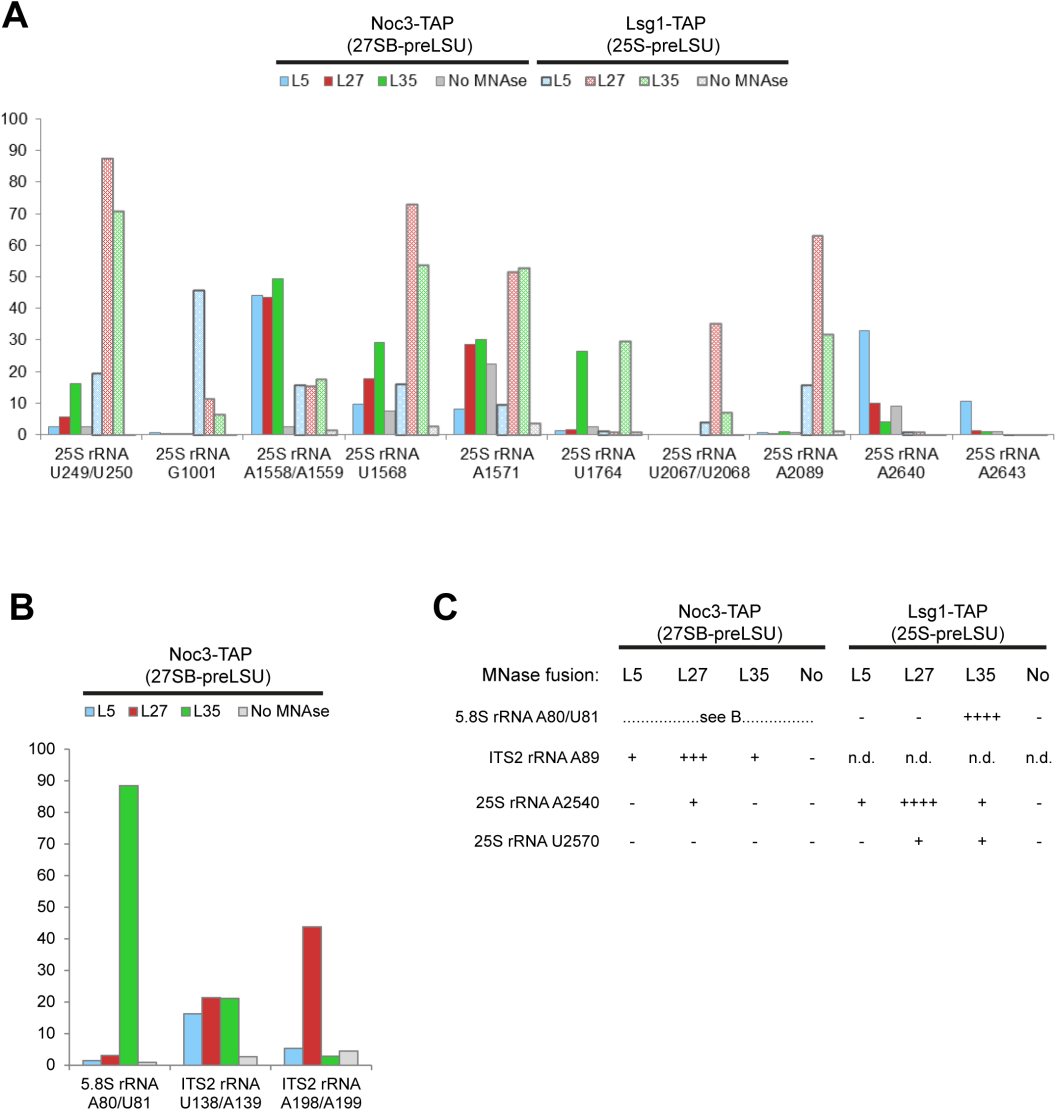
Tethered MNase cleavage positions in the rRNA sequence as analyzed by primer extension assays. 27SB-preLSUs and 25S-preLSUs were affinity purified via Noc3-TAP or Lsg1-TAP from yeast strains expressing no MNase (No) or MNase fused to rpL5 (L5), rpL35 (L35) or rpL27 (L27). rRNA 5’ ends generated after activation of MNase were analyzed by random primer extension assays and high throughput sequencing or by targeted primer extension assays (see Materials and Methods). In (A) and (B) percent of cleavage (TCR) at the indicated rRNA positions (+/- one nucleotide) was estimated based on high throughput sequencing data. Relative cleavage efficiency was estimated in (C) by inspection of targeted primer extension data shown in S4, S10 and S12 Figs as very strong (++++), strong (+++), weak (++), very weak (+) or not detectable (-).

High TCR values (between 30% and 100%) were observed in all experimental datasets in regions of the expected pre-rRNA 5’ ends of 27SB-preLSUs and 25S-preLSUs and around sites of 1 -methyladenosine and 3-methyluridine modifications which are known to inhibit the progression of reverse transcriptases (S3 Fig). High TCR values were also observed outside these regions (> 2 nucleotides away) for a number of sites (summarized in Fig 3A and 3B, detailed information in S4–S12 Figs) in LSU precursors purified from strains expressing MNase fusion proteins. These TCR values significantly exceeded the estimated local noise and the equivalent TCR values of particles purified from strains not expressing MNase fusion proteins (>5 times, respectively). Targeted primer extension reactions with primers hybridizing at selected positions which were read out by capillary electrophoresis (data not shown) and by gel electrophoresis confirmed the positions and relative efficiency of these termination sites which we interpret as MNase cleavage sites (S4–S12 Figs). In fact, due to a size cutoff step for cDNAs analyzed in the high throughput sequencing approach we could determine the position and approximate frequency of three MNase cleavage sites only by targeted primer extension analyses (summarized in Fig 3C). Neither targeted primer extension analyses nor the high throughput sequencing approach indicated any major MNase cleavage sites in the 119 nucleotide long 5S rRNA (data not shown).

**Fig 4:**
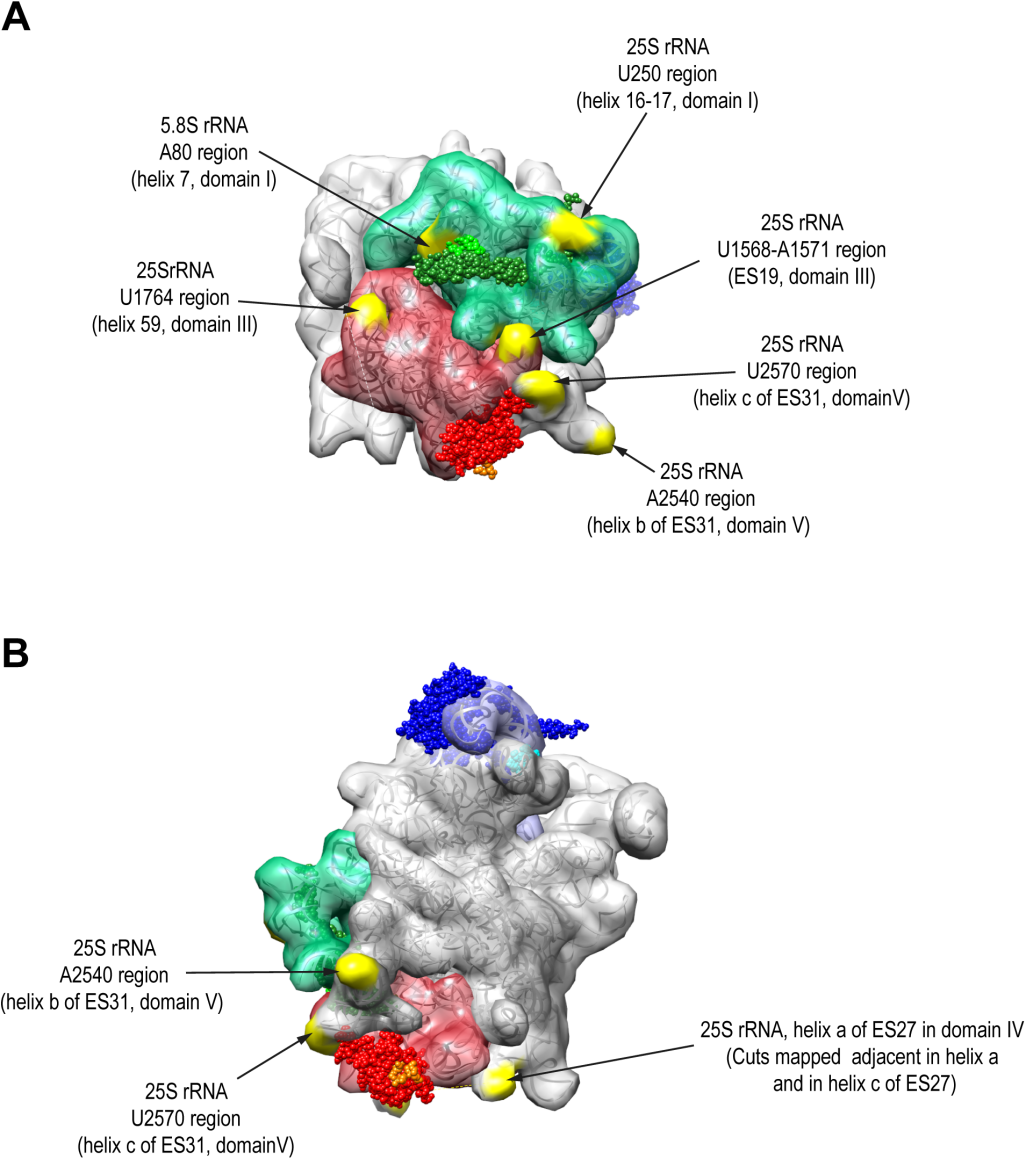
Location of rpL27-MNase and rpL35-MNase cleavages in the 25S-preLSU rRNA tertiary structure. Structures of rRNA, rpL5, rpL27 and rpL35 in 25S-preLSUs (pdb file 5apo, [12]) are visualized using the same color scheme as described in Fig 1, with rRNA cleavage sites highlighted in yellow. In the orientation shown in (A) LSU rRNA domains I and III are in the center, in the orientation shown in (B) the subunit interface side.

**Fig 5:**
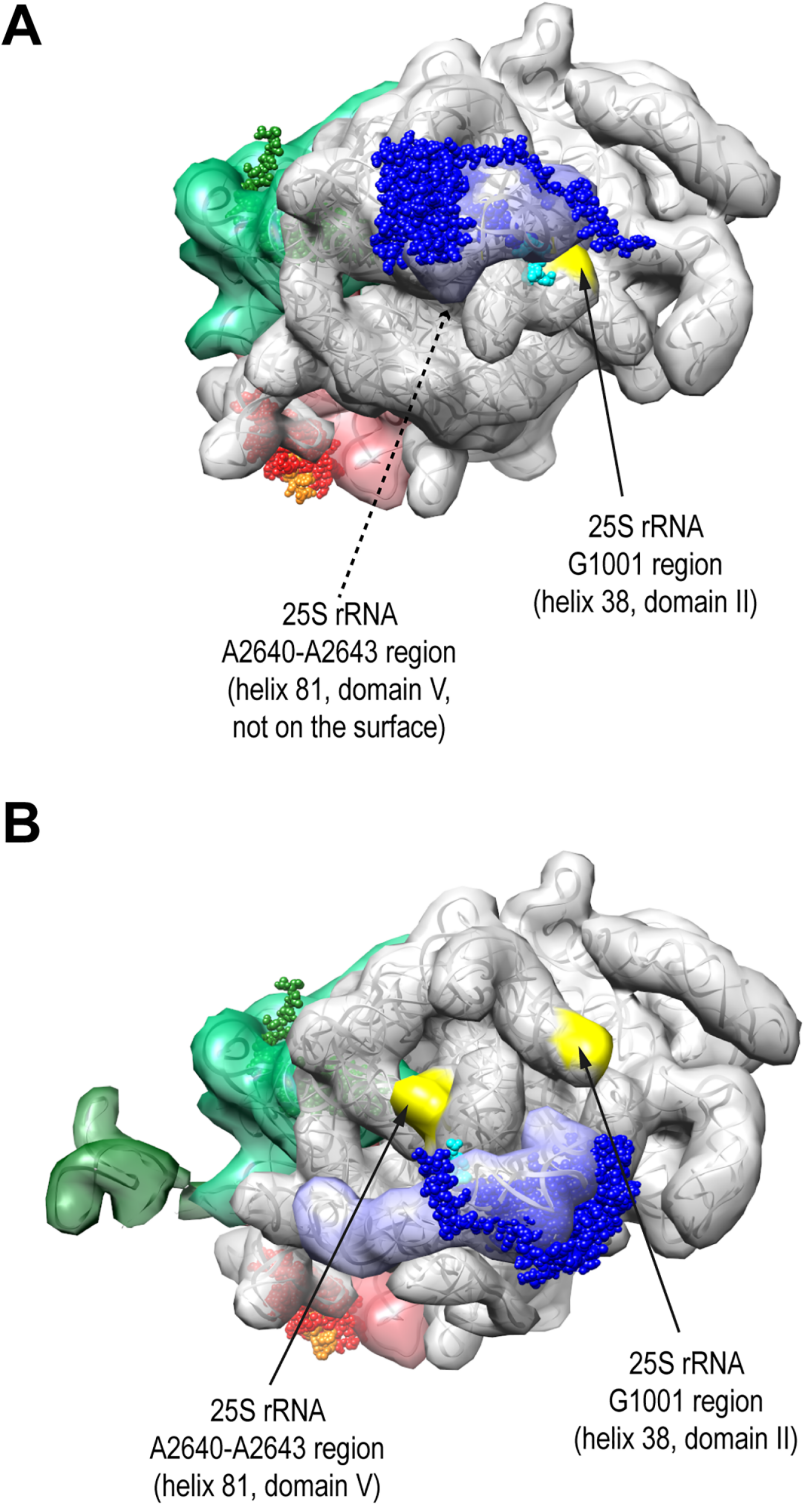
Location of rpL5-MNase cleavages in 25S-preLSU and 7S-preLSU rRNA tertiary structure models. Structures of rRNA, rpL5, rpL27 and rpL35 in 25S-preLSUs (A) or 7S-preLSUs (B) are shown using the color scheme as in Fig 1, with rRNA cleavage sites at the surface highlighted in yellow. The orientation shown is centered on the central protuberance. The pdb files 5apo and 3jct used to create the Figure were published in [12,16].

We analyzed the specificity and distribution of the identified cleavage sites in 25S-preLSUs with the help of primary, secondary and tertiary structure data currently available for these late cytoplasmic LSU precursors. In agreement with a previously reported sequence bias of MNase activity on DNA substrates [35], cleavages were found nearly exclusively 5’ of uracil or adenine bases. Moreover, all cleavage sites in 25S-preLSUs were found in single stranded regions, either stem-loops or internal loops (S4A–S12A Figs). Preferential cuts by each of the three ribosomal protein fusions indicated that tethering of MNase to specific ribosomal regions added another layer of substrate selectivity. Indeed, locations of the cuts in a structure model of 25S preLSUs revealed that cleavage efficiencies at the various sites correlated with spatial proximity of the respective fusion proteins (Fig 4). Sites preferentially cleaved by both MNase-rpL27 and MNase-rpL35 clustered in their proximity in LSU rRNA domains I and III and in neighboring parts of LSU rRNA domain V. In good correlation with the known binding site within the ribosome MNase-rpL27 fusions showed higher preference for sites oriented towards the subunit interface (Figs 3 and 4, 25S rRNA U250 region, U1568-A1571 region, U2570 region and A2540 region) while only MNase-rpL35 fusions efficiently cleaved at two sites at the opposite side of the ribosomal area formed by LSU rRNA domains I and III (Figs 3 and 4, 5.8S rRNA A80 region and 25S rRNA U1764 region). Near exclusive cuts in 25S rRNA helix 38 around nucleotide 1001 were found in 25S preLSUs with MNase tethered to rpL5 (Fig 3A, 25S rRNA G1001 region). As shown in Fig 5A, this site is directly located next to the N-terminal domain of rpL5 in the LSU central protuberance. Major MNase-rpL5 dependent cleavages in cytoplasmic mature 80S ribosomes were previously mapped in adjacent single stranded regions in helix 38 at positions 1014 and 1026 [21] with only minor cutting events observed at position 1001. Direct access to position 1001 seems partially hampered by the adjacent 5S rRNA in yeast 80S ribosomes [18]. In agreement with previous work, we take these observations as indication for changes in the spatial orientation of helix 38 during late steps of cytoplasmic LSU maturation and the formation of 80S ribosomes [13,14]. In addition, we conclude from the combined results that RNA cleavage events in tethered MNase assays can be read out by targeted and high-throughput RT reactions to faithfully characterize rRNA secondary and tertiary structure features in specific regions of LSU precursors.

### Structural transitions at the LSU rRNA domain I and III interface region, the central protuberance and in expansion segment ES27 during maturation of 27S-preLSUs to 25S-preLSUs

Comparison of cleavage efficiencies observed in 25S-preLSUs with the ones in 27SB-preLSUs indicated for some sites similar and for other sites diverging accessibility for MNase fusion proteins. Cleavages by MNase-rpL35 tethered to LSU-rRNA domain I (via rpL35) at proximal sites in LSU rRNA domain I in the 5.8S rRNA and in LSU rRNA domain III were observed with similar intensities in 25S-preLSUs and in 27SB-preLSUs (Figs 3 and 4, 5.8S rRNA A80 region and 25S rRNA U1764 region, S4 and S8 Figs). We take this as indication for an advanced assembly state of rpL35 in 27SB-preLSUs and for completion of major aspects of the LSU rRNA domain I and III configuration. In contrast, MNase-rpL27 and MNase-rpL35 cleavage intensities clearly changed in the subarea of LSU rRNA domains I and III which is close to the subunit interface. Here, the ITS2 spacer emanates and helices 76–79 and expansion segment 31 (ES31) of LSU rRNA domain V join. Accessibility in that region around nucleotides 2540 (helix b of ES31) and 2570 of 25S rRNA (helix c of ES31) was strongly reduced and similar effects were observed for cleavages around nucleotide 250 of 25S rRNA (internal loop between helices 16 and 17) (Fig 3, S5 and S10 Figs). Moreover, in the surrounding area lower accessibility in 27SB-preLSUs around nucleotide 1571 of 25S rRNA (ES19) in LSU rRNA domain III was observed (Fig 3, S7 Fig). Concomitantly, apparent cleavage intensities by all three nuclease fusions increased in the adjacent internal loop at nucleotide position 1558 of ES19 (Fig 3, S7 Fig). We take these observations as evidence for substantial differences between the spatial organization of 27SB-preLSUs and 25S-preLSUs in the region where the ITS2 spacer emanates. In part this premature organization of 27SB-preLSUs is still reflected in cryo-electron microscopy structures of downstream 7S-preLSUs [16]. Here, two early recruited biogenesis factors (Rlp7, Nop7) partially cover 25S rRNA nucleotides 2570 and 1571. By contrast, the decreased accessibility of the 25S pre-rRNA regions around nucleotides 2540 and 250, and the high accessibility of the region around 1558 are not explained by the spatial organization seen in 7S-preLSU structure models. Substantial changes in this region during maturation from 27SB to 7S-preLSUs are furthermore supported by the results of previous biochemical analyses indicating a concomitant significant increase in binding strength of the two local ribosomal proteins rpL2 and rpL43 [17].

Two other regions were identified for which tethered MNase cutting efficiencies significantly changed in 27SB-preLSUs. Accessibility in 27SB preLSUs for MNase fused to rpL27 diminished in helices b and c of the expansion segment 27 (ES27) (Fig 3 and S9 Fig, location of ES27 in Fig 4B) which was observed in several alternative orientations in mature ribosomal populations [36]. Apart from this, cleavage of MNase-rpL5 in helix 38 at position 1001 was strongly reduced in 27SB-preLSUs. At the same time new cuts were detected for this fusion protein in the stem-loop of helix 81 around nucleotides 2640 and 2643 in 27SB-preLSUs which could not be observed in 25S-preLSUs (Fig 3, S11 Fig). This region is completely covered by rRNA in structure models of mature LSUs and 25S-preLSUs but it is well accessible and in close proximity to the N-terminal domain of rpL5 in 7S-preLSUs as a consequence of the rotated state of the central protuberance (Fig 5) [12,14,16,18]. We take these results as evidence for a substantial proportion (> 40% total cleavage in helix 81, Fig 3) of 27SB-preLSUs having the central protuberance in an immature folding state similar to the one previously observed for 7S-preLSUs.

### Folding of the ITS2 spacer region in 27SB-preLSUs

**Fig 6:**
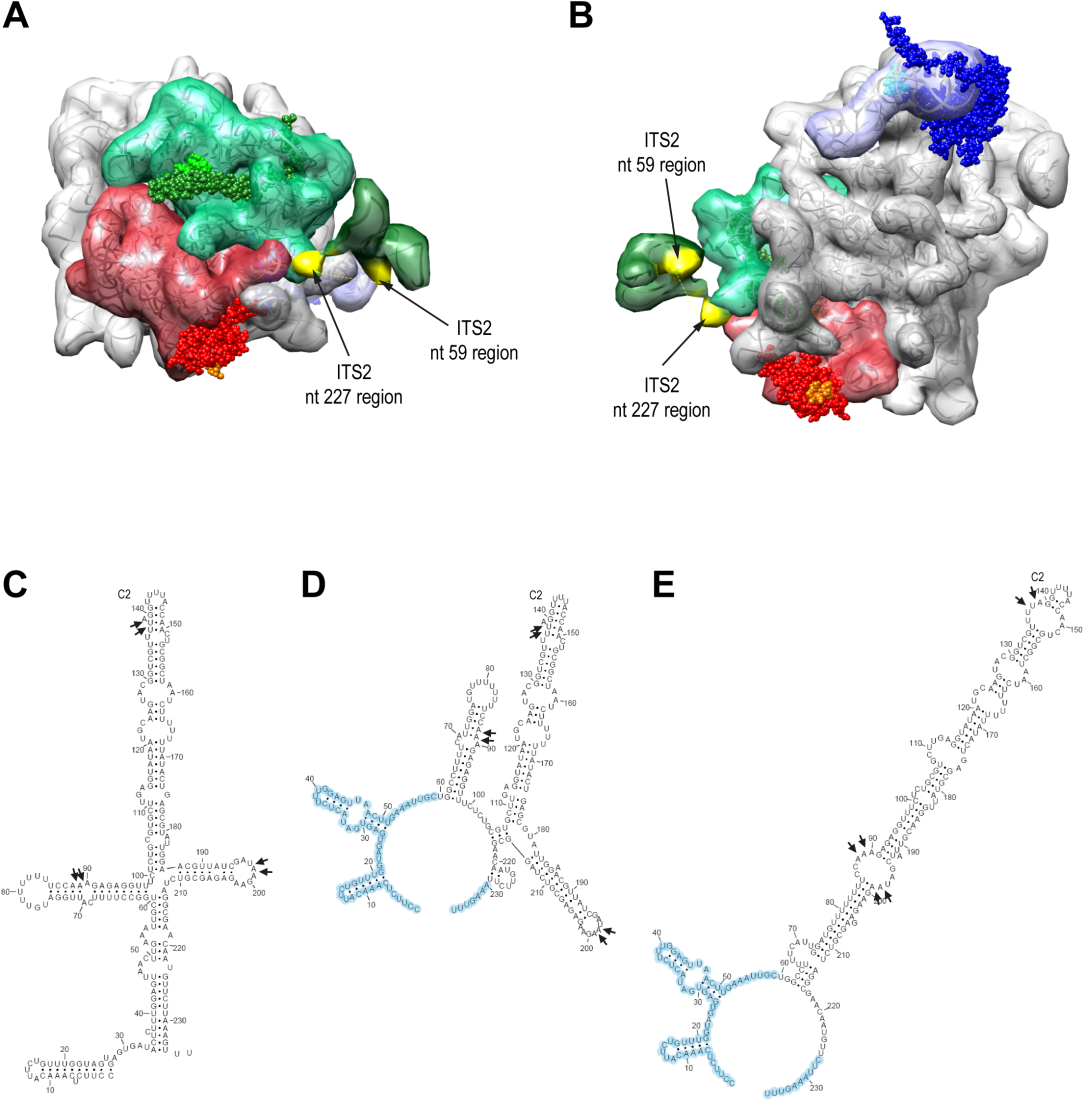
Location of tethered MNase cleavages in secondary and tertiary structure models of the ITS2 rRNA region. Structures of rRNA, rpL5, rpL27 and rpL35 in 7S-preLSUs are shown using the same color scheme as described in Fig 1, centering on LSU rRNA domains I and III in (A) and on the subunit interface side in (B). The last nucleotides of the terminal ITS2 region resolved in the 7S-preLSU structure model are colored in yellow. The pdb file 3jct used to create the figures (A) and (B) was published in ([16]). In (C-E) three secondary structure models of the ITS2 region are shown which were taken from [29](C), [30](D) and [31](E). In (D) and (E) the terminal parts of the ITS2 region with a secondary structure matching the one observed in cryo electron microscopy analyses of 7S-preLSUs shown in (A) and (B) are highlighted in blue. Position of the endonucleolytic C2 processing site (C2) according to [28,49] and tethered MNase cleavage sites (black arrows) are indicated.

**Fig 7:**
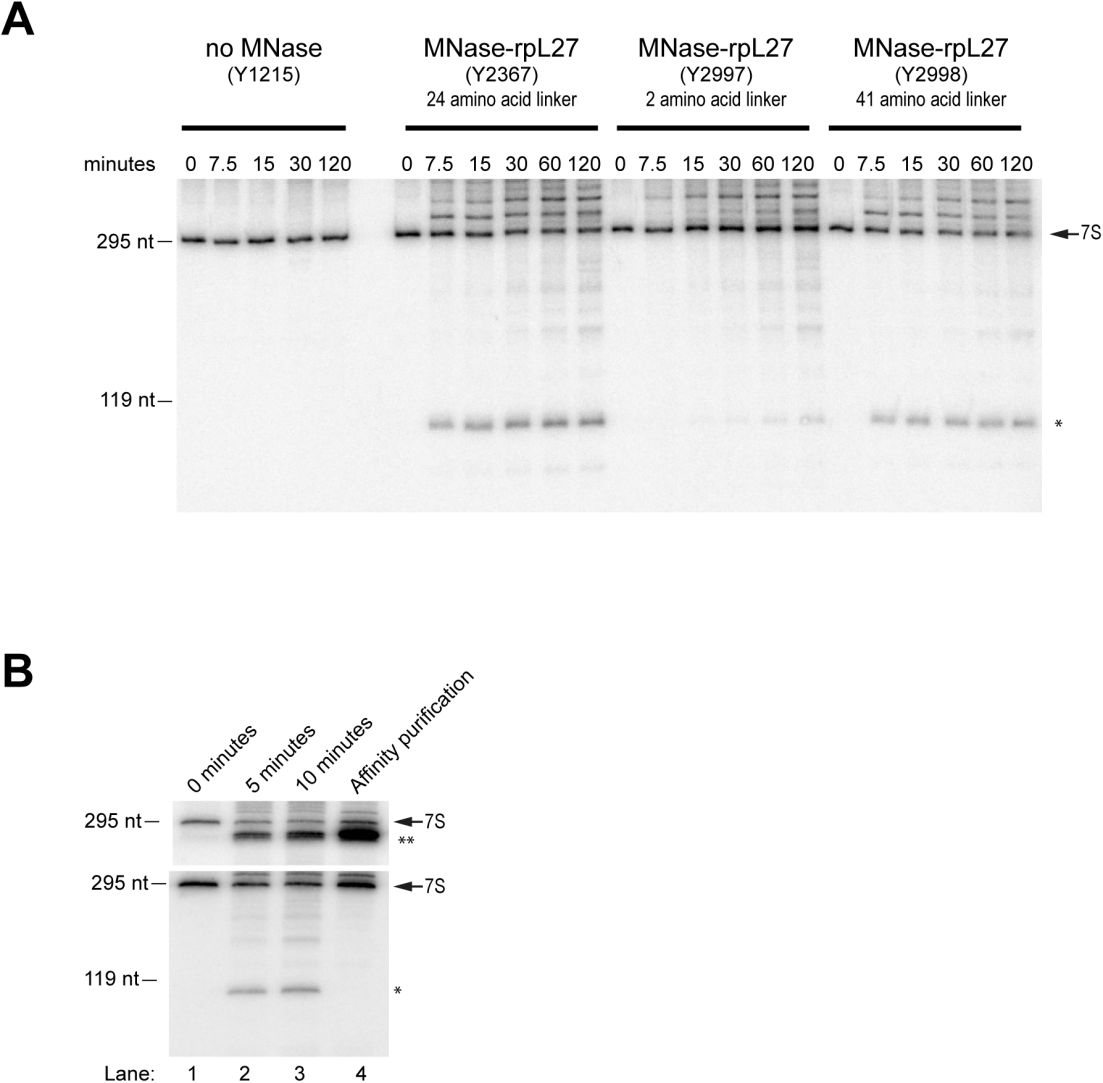
Release of the central ITS2 fragment from 27SB-preLSUs after site specific ITS2 cleavage by MNAse tethered to rpL27. In (A) calcium dependent production of the central ~110nt ITS2 fragment was analyzed in cellular extracts of the indicated strains expressing MNase fused to rpL27 with a linker of 2, 24 and 41 amino acids. Extracts were incubated at 16°C for the indicated times in the presence of calcium and RNA was analyzed by Northern blotting using probe #O442 (see Materials and Methods). The central ~110nt ITS2 fragment is marked by an asterisk. In (B) the cellular extract of a strain expressing MNase fused to rpL27 was incubated for the indicated times at 16°C in the presence of calcium (lanes 1–3) and after ten minutes of incubation LSU precursors were affinity purified via Noc3-TAP (lane 4, see Materials and Methods). RNA was analyzed by Northern blotting using probe #O210 in the upper panel and probe #O442 in the lower panel. The central ~110nt ITS2 fragment is marked by a star in the lower panel and a neighboring fragment spanning the 5.8S rRNA region and the 5’ region of ITS2 is marked by two stars in the upper panel. Running behavior of RNA fragments of defined length are indicated on the left.

The tertiary structure of terminal ITS2 nucleotides 1 to 59 and 230 to 237 in purified yeast 7S-preLSUs could be recently resolved by cryo-electron microscopy [16]. In addition, secondary structure probing experiments, phylogenetic analyses and free energy calculations led to alternative secondary structure models for the yeast ITS2 rRNA region, including the central part spanning nucleotides 61 to 229 (Fig 6C–6E) [29–31]. We observed two cuts in the ITS2 region around nucleotides 89 and 198 in 27SB-preLSUs purified from strains expressing the MNase-rpL27 fusion (Fig 3B and 3C, S12 Fig). Less efficient cleavage was detected in the ITS2 close to the processing site C2 around nucleotide 138 in 27SB-preLSU populations from strains expressing any of the three utilized MNase fusions (Fig 3B, S12 Fig). In only one of the proposed ITS2 secondary structure models all of the identified cleavages fall into single stranded regions, the preferred substrate for the MNase fusions (Fig 6C–6E). Here, the two cuts specific for MNase-rpL27 are at opposite sites of an internal loop in the center of a long helical structure (termed helix III) spanning ITS2 nucleotides 62–216 (Fig 6E). Concurrent cleavage at these two positions could therefore release the ITS2 rRNA central region from 27SB-preLSUs whereas according to the other models it would be retained by base-pairing with the ITS2 terminal regions. Indeed, substantial amounts of the central ITS2 fragment with the expected size were detected in purified 27SB-preLSUs after activation of the MNase-rpL27 fusion (Fig 2E). Calcium triggered production of the central ITS2 fragment was also observed in total cellular extracts and critically depended on the size of the linker between MNase and rpL27 (Fig 7A). Affinity purification of cleaved LSU precursors via Noc3-TAP from respective calcium treated extracts showed that after cleavage at positions 89 and 198 by MNase-rpL27 the central ITS2 fragment is released from 27SB-LSUs while flanking regions spanning the 5.8S rRNA are still associated (Fig 7B). Hence, the behavior of the central ITS2 fragment matches predictions made based on the secondary structure model shown in Fig 6E, but is not explained by the models shown in 6C and D. We take this as further evidence for folding of the ITS2 central region in a long helical structure with interspersed internal loops in a significant 27SB-preLSU population. Furthermore, the observed impact of the ribosomal position of tethered MNase probes and of the linker length between MNase and rpL27 on cleavages in the ITS2 central region can serve as indication for the spatial orientation of this helix. Accordingly, the distal part of the stem with the C2 processing site is rather flexible in orientation while location of the proximal part is more restricted to an area including the vicinity of rpL27.

## Materials and methods

### Yeast strains, plasmid construction and microbiological procedures

Transformation of yeast cells and their cultivation in full or synthetic medium were done according to standard procedures [37,38]. Yeast strains expressing chromosomally-encoded TAP-tagged LSU biogenesis factors Noc1, Noc3, or Lsg1 were created as described in [39]. Details about oligonucleotides, plasmids and yeast strains used in this study can be found in S1 –S3 Tables, respectively. Construction of plasmids was done following standard procedures [40]. Yeast strain Y1082 was described in [41], yeast strain Y1215 in [17], plasmid K375 in [42], plasmid K855 in [41], plasmid pBS1539 in [39], plasmid Yeplac181 in [43] and plasmids K1735, K1742, K1743, K1940, and K1941 in [21]. Apparent generation times in YPD (1% (w/v) yeast extract, 2% (w/v) bacto peptone, 2% (w/v) glucose) at 30°C of yeast strains expressing tagged factors and r-proteins were determined using a TECAN reader (TECAN) as described in [21] and are listed in S3 Table.

### Affinity purification of LSU precursor populations and subsequent activation of MNase fusion proteins

Yeast strains Y2592, Y2593, Y2598, Y2599, Y2601, Y2663, Y2664, Y2795, Y3389, Y3397, Y3405 and Y3413, were cultivated in YPD to an optical density between 0.8 and 1.2. Cells from 6 liters of culture (Noc1-TAP and Noc3-TAP strains) or from 2 liters of culture (Lsg1-TAP strains) were then harvested by centrifugation at room temperature (3000g) and were washed in ice cold water and then in 15 milliliter ice cold buffer AG100 (100 mM potassium chloride, 20 mM Tris pH8, 5 mM magnesium acetate, 5 mM EGTA pH8, 1 mM PMSF, 2 mM Benzamidine). To each gram of wet cell pellet 1.5 milliliter of cold buffer AG100 with 0.04 Units/microliter RNAsin (Promega) was added and the resulting suspension was distributed in portions of 0.8 milliliter to reaction tubes (2 milliliter volume) loaded with 1.4 gram of glass beads (0.75–1 millimeter, Roth). The tubes were rigorously mixed on a vibrax mixer (IKA) at 4°C and maximum speed for three times 8 minutes with cooling on ice in between. The extract was cleared from cell debris and glass beads by two consecutive centrifugation steps for 5 minutes at 13000g and 4°C and transfer of the respective supernatants to new reaction tubes. Protein concentrations of the clarified extracts were determined by Bradford protein assay (Bio-Rad) and a volume containing 300 microgram protein was taken off for RNA analyses (see below, RNA was dissolved in 30 microliter of water). Triton X-100 and Tween 20 were added to final concentrations of 0.5% (w/v) and 0.1% (w/v), respectively, and the extracts were loaded on 150 microliter IgG sepharose beads (GE Healthcare) equilibrated in buffer AG100+ (AG100 adjusted to 0.5% (w/v) Triton X-100 and 0.1% (w/v) Tween 20). After batch incubation at 4°C for 1 hour the affinity matrix was transferred into 15 milliliter plastic columns (Poly Prep chromatography columns, 0.8 x 4 centimeter, Bio-Rad) and washed twice with 2 milliliter of ice cold AG100+ and once with 10 milliliter of AG100+. The matrix was transferred in 0.5 milliliter of ice cold AG100 into a 1.5 milliliter reaction tube, 10% of the suspension was taken off for RNA analyses (see below, RNA was dissolved in 10 microliter of water), and after centrifugation for 2 minutes at 2000g at 4°C the volume was reduced to 200 microliter by removing some of the supernatant. Calcium chloride was added to the suspension to a final concentration of 8mM and after 10 minutes incubation at 16°C RNA was extracted (see below, RNA was dissolved in 60 microliter of water).

### Activation of MNase fusion proteins in cellular extracts

Yeast strains Y1215, Y2367, Y2997 and Y2998 were cultivated in 400 milliliter of YPD at 30°C. Cellular extracts were prepared in buffer AG100 as described above. A volume of the extract containing 300 microgram protein was taken off for RNA analyses (see below, RNA was dissolved in 30 microliter of water). The extract was then adjusted to 8 mM of calcium chloride, incubated at 16°C for the respective periods of time and a volume of the extract containing 300 microgram protein was taken off for RNA analyses (see below, RNA was dissolved in 30 microliter of water).

### Activation of MNase fusion proteins in cellular extracts and subsequent affinity purification of LSU precursors

Yeast strain Y2599 was cultivated in 400 milliliter of YPD at 30°C. Cellular extracts were prepared in buffer AG100 as described above. A volume of the extract containing 300 microgram protein was taken for RNA analyses (see below, RNA was dissolved in 25 microliter of water). The extract was then adjusted to 8 mM of calcium chloride, incubated at 16°C for 10 minutes and again a volume of the extract containing 300 microgram protein was taken off for RNA analyses (see below, RNA was dissolved in 20 microliter of water). Triton X-100 and Tween 20 were added to the residual extract to final concentrations of 0.5% (w/v) and 0.1% (w/v), respectively, and concentration of EGTA was adjusted to 10mM. The extracts were then loaded on 100 microliter IgG sepharose beads (GE Healthcare) equilibrated in buffer AG100+. After batch incubation at 4°C for 1 hour the affinity matrix was transferred into 15milliliter plastic columns (Poly Prep chromatography columns, 0.8 x 4 centimeter, Bio-Rad) and washed twice with 2 milliliter of ice cold AG100+ and once with 10 milliliter of AG100+. The matrix was transferred in 0.5 milliliter of ice cold AG100 into a 1.5 milliliter reaction tube and after centrifugation for 2 minutes at 2000g at 4°C the supernatant was carefully removed. RNA was then extracted from the IgG matrix (see below, RNA was dissolved in 20 microliter of water).

### RNA extraction

RNA was extracted by hot acidic phenol–chloroform treatment as previously described [44] with the following modifications. Fractions taken for RNA analyses were added to 500 microliter of ice cold buffer AE+ (50mM sodium acetate pH 5.3, 20mM EDTA), 50 microliter of 10% SDS and 500 microliter of phenol (equilibrated in 50mM sodium acetate pH 5.3, 10mM EDTA). After 6 minutes of rigorous shaking at 65°C each sample was cooled on ice for two minutes and then centrifuged at 13000g and 4°C for two minutes. The supernatant was transferred to a new reaction tube containing 500 microliter of phenol (equilibrated in 50mM sodium acetate pH 5.3, 10mM EDTA) and after vortexing for 10 seconds the mixture was again centrifuged at 13000g and 4°C for two minutes. This procedure was repeated once with 500 microliter chloroform. 300 microliter of the supernatant was then transferred to a reaction tube containing 750 microliter of ethanol to which 30 microliter of 3M sodium acetate pH 5.3 were added. In case of affinity purified fractions 2 microliter of a 5 milligram per milliliter glycogen solution were added. Each sample was then briefly vortexed and incubated for more than 10 minutes at -20°C before centrifugation for 30 minutes at 4°C and 13000g. The supernatant was carefully discarded and the pellet of affinity purified fractions was dissolved in the volume of ice cold water indicated above.

### Northern blotting

5 microliter and 4 microliter of extracted RNA were separated on 1.5% formaldehyde/MOPS agarose gels and on 8% polyacrylamide/urea/TBE gels and transferred to nylon membranes (MP Biomedicals) as described in [40]. Hybridization with P32- end labelled oligonucleotide probes (see below) indicated in the figure legends was performed in 50% formamide/5x SSC/0,5% SDS/5x Denhardt’s solution at 30°C. Washing was done for 15 minutes in 2xSSC and for 15 minutes in 1xSSC. Image plates (FujiFilm) were exposed to the radioactive signals on the washed blots and then read out using a phosphor imager device (FLA3000, FujiFilm).

### Targeted primer extension analyses

Targeted primer extension analyses were done with oligonucleotide primers indicated in the figure legends and listed in S1 Table as described in [45] with some modifications. In brief, 50 picomol of oligonucleotides were end labeled with T4 PNK (NEB) according to the manufacturers instruction in 15 reaction volume using 0.050 mCi [gamma-P32]Adenosine 5'-triphosphate (Hartmann Analytic, 10 mCi / milliliter). The reaction was terminated by addition of 1 microliter of 0.5M EDTA and 30 microliter of water and the labeled oligonucleotides were purified using Micro Biospin 6 size exclusion columns (Bio-Rad). 2 microliter of labeled primer was incubated with RNA from affinity purified fractions (1 microgram as determined by UV spectrophotometry) and 1 microliter of 10mM dNTP mix (NEB) in a volume of 13 microliter for 5 minutes at 65°C in a thermocycler (PCR Sprint, Thermo Electron Corporation). After cooling down to 4°C primer extension reactions were performed for 90 minutes at 46°C in 20 microliter reaction volume using Superscript III according to the manufacturer’s instructions. RNA was hydrolyzed by addition of 2.5 microliter of 1M sodium hydroxide and 0.5 microliter of 0.5M EDTA at 56°C for 30 minutes. Subsequently, 2.5 microliter of 1M hydrogen chloride, 2 microliter of a 2 milligram / milliliter glycogen solution, 12.5 microliter of 7.4M ammonium acetate and 100 microliter ethanol were added. After brief mixing each sample was incubated over night at -20°C and then centrifuged for 10 minutes at 4°C and 13000g. The supernatant was carefully discarded and the pellet was washed with 100 microliter ice cold 70% (v/v) ethanol. After drying of the pellet for 3 minutes at 80°C the cDNA was dissolved in 10 microliter of a 1:2 diluted solution of 95% deionized formamide, 20mM EDTA pH8, 0.02% (w/v) bromophenol blue and xylene cyanol. Sequencing reactions using the same primers and a plasmid containing the yeast 35S rRNA gene (K375) were performed with the Thermo Sequenase cycle sequencing kit (785001KT Affymetrix) following the manufacturer’s instructions. Products of sequencing reactions and of primer extension analyses were incubated for 3 minutes at 75°C, cooled on ice and then size separated on a 6% polyacrylamide/urea/taurin gel according to the instruction provided in the Thermo Sequenase cycle sequencing kit. Running time was adapted to optimally resolve the size range of interest for each of the respective primer extension reactions. After drying of the gel on filter paper (Cellulose, Roth) on a vacuum gel dryer at 80°C radioactive signals were read out using a FLA3000 (FUJI).

### Random primer extension analyses and high throughput sequencing

Sequencing libraries were prepared as previously described [34] using 1 microgram of RNA and oligonucleotide #O3955 as random primer and #O3954 as adapter. During PCR amplification oligonucleotide #O3956 was used as forward primer and oligonucleotides #O3957, #O3958, #O3959, #O3960, #O4032, #O4033, #O4187, #O4188 as indexing primers. The expected size distribution of the amplified sequencing libraries was confirmed using the High Sensitivity DNA kit (Agilent Technologies) on a 2100-Bioanalyzer capillary electrophoresis system. Paired end sequencing (2x 80 nt) was done on a Illumina Miseq system (Illumina). Raw data are available at the Gene Expression Omnibus (https://www.ncbi.nlm.nih.gov/geo/, accession number: GSE97276). Mapping of reads on the S.cerevisiae rDNA region (strain S288c) was done using Bowtie 2 [46] with standard parameters. Before mapping, 7 nucleotide random barcodes on the 5‘ ends of reads 1 were removed and reads 1 and 2 with a “AGATCGG” sequence at a distance of more than 20 nucleotides from their 5’ end were trimmed at this site. For each dataset 300.000 mapped reads with an insert size between 75 and 750 nucleotides and a Bowtie 2 quality score of Q> = 40 were selected for further statistical evaluation. Bias introduced during PCR amplification was estimated and corrected as described [34] making use of the random barcode in the adapter primer #O3954. Accordingly, for each experimental condition reads were grouped based on identical mapped start site and length and in each group the number of observed unique random barcodes was determined. This number was then further corrected by taking into account possible ligation of cDNA fragments of one of the groups with adapters with identical barcodes. The likelihood of these ligation events was estimated based on the amount of different random barcodes encoded in the adapter primer and the observed frequency of adenine, guanine, cytosine or thymine at each barcode position in the mapped reads [34]. The resulting estimated unique counts for cDNA fragments with defined start and stop positions were used to calculate for each position in LSU pre-rRNA the coverage and the termination to coverage ratio (TCR). Calculated TCR values for each experimental condition are given in S4 Table. Termination ratios shown in Fig 3 and S3 Fig were calculated as one minus the product of the TCR value at the indicated nucleotide positon and the direct neighbors (+/- 1 nucleotide) to account for possible nucleotide trimming or addition reactions during the experimental procedure. To estimate the noise of random stops of primer extension reactions at each pre-rRNA position for each experimental sample 100 virtual datasets were created in which the pool of observed fragment start sites was randomly combined with the pool of observed fragment lengths. Coordinates of fragments ending at sites with high TCR values (>15%) were kept as observed in the experimental dataset. The averaged coverage distribution of the virtual datasets matched strikingly well the one observed in the experimental dataset (coefficient of determination R2 > 0.98 for all datasets) thus indicating that selectivity in priming sites together with the fragment length distribution are major factors influencing the local noise of termination events. The local noise was then estimated as the maximum TCR within a region of 5 nucleotides of all virtual datasets derived from either Lsg1-TAP or Noc3-TAP particles. All data processing and visualization was done with VBA scripts in the Excel (Microsoft) environment (scripts are available upon request).

### Analysis and visualization of pre-ribosomal secondary and tertiary structure models

Ribosomal structure models were obtained from the Research Collaboratory for Structural Bioinformatics Protein Data Bank (RCSB-PDB, http://www.rcsb.org/pdb/home/home.do). Pdb files used are indicated in the legends to the respective Figures. They were analyzed and visualized with the UCSF Chimera software package [47]. Secondary structure models of rRNA were visualized using RnaViz [48].

## Supporting information

S1 Fig Positions of probes used for northern blotting and primer extension analyses. In the upper panel positions of the oligonucleotide probes used in this study (black bars) and of major processing sites (arrows) in yeast pre-rRNA are indicated. Below, rRNA precursors predominantly contained in the pre-LSU populations relevant for this study are shown. Mature rRNA regions are represented by grey bars and spacer sequences by a black line which is dotted in case that partial trimming can occur.
(TIF)

S2 Fig Northern blotting analyses of affinity purified LSU precursors. 27SA-preLSUs (A), 27SB preLSUs (B) and 25S preLSUs (C) were affinity purified via Noc1-TAP, Noc3-TAP and Lsg1-TAP respectively from cellular extracts of strains expressing no MNase (lanes 2 and 6), MNnase fused to rpL5 (lanes 1 and 5), to rpL27 (lanes 4 and 8) or to rpL35 (lanes 3 and 7). RNA from total cellular extracts (lanes 1–4) and affinity purified fractions (lanes 5–8) was analyzed by northern blotting with the probes indicated on the left in (A-C).
(TIF)

S3 Fig Major MNase independent primer extension stops in LSU rRNA. 27SB-preLSUs and 25S-preLSUs were affinity purified via Noc3-TAP or Lsg1-TAP from yeast strains expressing no MNAse (No) or MNAse fused to rpL5 (L5), rpL35 (L35) or rpL27 (L27). After activation of MNAse the positions of generated rRNA 5’ ends were analyzed by random primer extension assays read out by high throughput sequencing. Percent of termination in the indicated rRNA positions (+/- one nucleotide) was estimated as described in Materials and Methods. 3-methyl-uridine is abbreviated with m3U, 1–methyl-adenosine with m1A.
(TIF)

S4 Fig MNase fusion dependent cleavages in specific rRNA regions of 27SB-preLSUs and 25S-preLSUs. In (A) positions of cleavages are indicated (black arrows) in secondary structure models of the respective rRNA regions. Cleavages previously mapped in helix 38 of 80S ribosomes are indicated by red arrows in S6 Fig. In S4–S11 Figs in (B) results of targeted primer extension reactions using the indicated primers and RNA of 27SB-preLSUs (Noc3-TAP) or 25S-preLSUs (Lsg1-TAP) from strains expressing no MNase (-), or MNase in fusion with rpL5 (L5), rpL27 (L27) or rpL35 (L35) are shown. For each RNA preparation the volume used in the reactions with varying primers was kept constant. Sequencing reactions (lanes G, A, T and C) were performed using the respective primers as described in Materials and Methods. In (C) the termination to coverage ratio determined by random primer extension and high throughput-sequencing (see Material and Methods) is plotted for each nucleotide position in the relevant rRNA region. Nucleotide position 2634, which has a 3-methyluridine base modification, is colored in red at the top of the diagram in S11 Fig. Data were obtained for strains expressing no MNase (Wt), MNase in fusion with rpL5 (L5), rpL27 (L27) or rpL35 (L35). Local noise (Noise) in the high throughput readout of random primer extension reactions was estimated as described in Material and Methods.
(TIF)

S5 Fig MNase fusion dependent cleavages in specific rRNA regions of 27SB-preLSUs and 25S-preLSUs. See figure legends for S4 Fig.
(TIF)

S6 Fig MNase fusion dependent cleavages in specific rRNA regions of 27SB-preLSUs and 25S-preLSUs. See figure legends for S4 Fig.
(TIF)

S7 Fig MNase fusion dependent cleavages in specific rRNA regions of 27SB-preLSUs and 25S-preLSUs. See figure legends for S4 Fig.
(TIF)

S8 Fig MNase fusion dependent cleavages in specific rRNA regions of 27SB-preLSUs and 25S-preLSUs. See figure legends for S4 Fig.
(TIF)

S9 Fig MNase fusion dependent cleavages in specific rRNA regions of 27SB-preLSUs and 25S-preLSUs. See figure legends for S4 Fig.
(TIF)

S10 Fig MNase fusion dependent cleavages in specific rRNA regions of 27SB-preLSUs and 25S-preLSUs. See figure legends for S4 Fig.
(TIF)

S11 Fig MNase fusion dependent cleavages in specific rRNA regions of 27SB-preLSUs and 25S-preLSUs. See figure legends for S4 Fig.
(TIF)

S12 Fig MNase fusion dependent cleavages in the ITS2 rRNA region of 27SB-preLSUs. In (A) results of targeted primer extension reactions using the indicated primers and RNA of 27SB-preLSUs (Noc3-TAP) from strains expressing no MNase (-), or MNase in fusion with rpL5 (L5), rpL27 (L27) or rpL35 (L35) are shown. Sequencing reactions (lanes G, A, T and C) were performed with primers O3840 and O3910 as described in Materials and Methods. In (B) the termination to coverage ratio determined by random primer extension and high throughput-sequencing (see Materials and Methods) is plotted for the 3’ region of ITS2. Data were obtained for strains expressing no MNase (Wt), MNase in fusion with rpL5 (L5), rpL27 (L27) or rpL35 (L35). Local noise (Noise) in the high throughput readout of random primer extension reactions was estimated as described in Materials and Methods.
(TIF)

S1 Table Oligonucleotides used in this study. (XLSX)

S2 Table Plasmids used in this study. (XLSX)

S3 Table Yeast strains used in this study. (XLSX)

S4 Table TCR values calculated from results of random primer extension analyses. TCR values and estimates of local noise in termination events were calculated as described in Materials and Methods.
(XLSX)
