# Supplementary figures and images for: Structural transitions during large ribosomal subunit maturation analyzed by tethered nuclease structure probing in S. cerevisiae
Source: PLoS One. 2017 Jul 7;12(7):e0179405. doi: 10.1371/journal.pone.0179405 (PMC5501410; doi:10.1371/journal.pone.0179405)

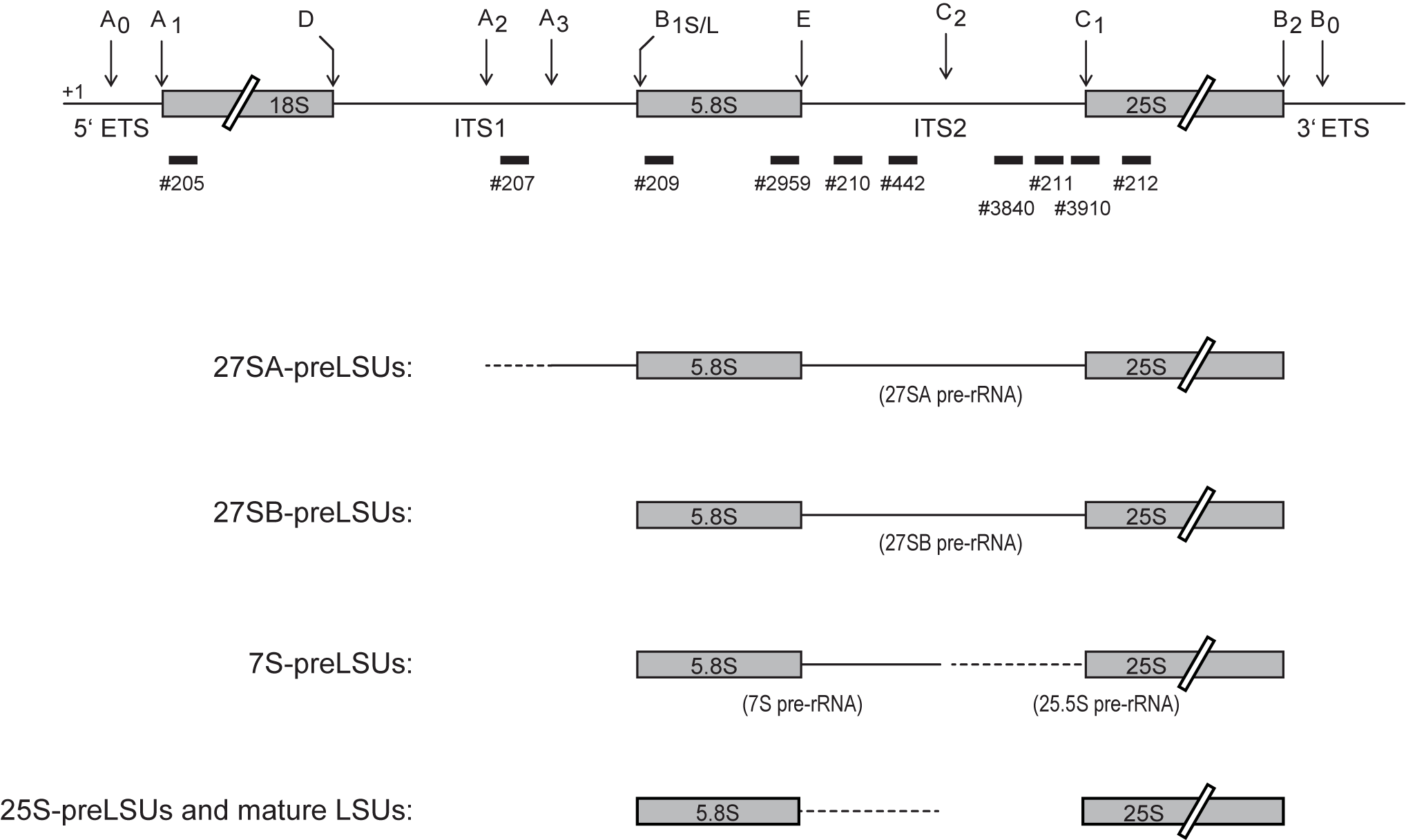

Supplement: S1 Fig — In the upper panel positions of the oligonucleotide probes used in this study (black bars) and of major processing sites (arrows) in yeast pre-rRNA are indicated. Below, rRNA precursors predominantly contained in the pre-LSU populations relevant for this study are shown. Mature rRNA regions are represented by grey bars and spacer sequences by a black line which is dotted in case that partial trimming can occur. (TIF) [file pone.0179405.s001.tif]

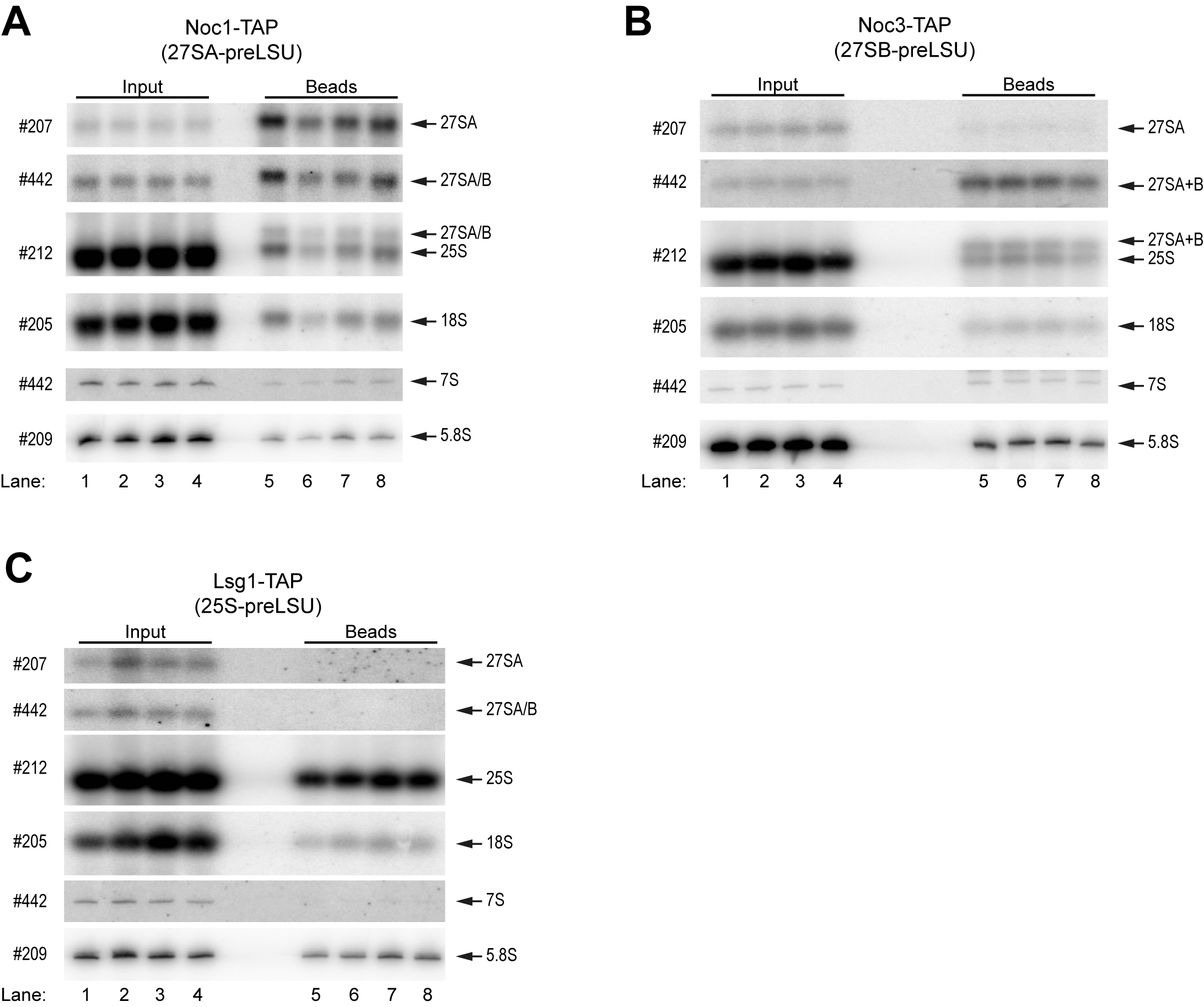

Supplement: S2 Fig — 27SA-preLSUs (A), 27SB preLSUs (B) and 25S preLSUs (C) were affinity purified via Noc1-TAP, Noc3-TAP and Lsg1-TAP respectively from cellular extracts of strains expressing no MNase (lanes 2 and 6), MNnase fused to rpL5 (lanes 1 and 5), to rpL27 (lanes 4 and 8) or to rpL35 (lanes 3 and 7). RNA from total cellular extracts (lanes 1–4) and affinity purified fractions (lanes 5–8) was analyzed by northern blotting with the probes indicated on the left in (A-C). (TIF) [file pone.0179405.s002.tif]

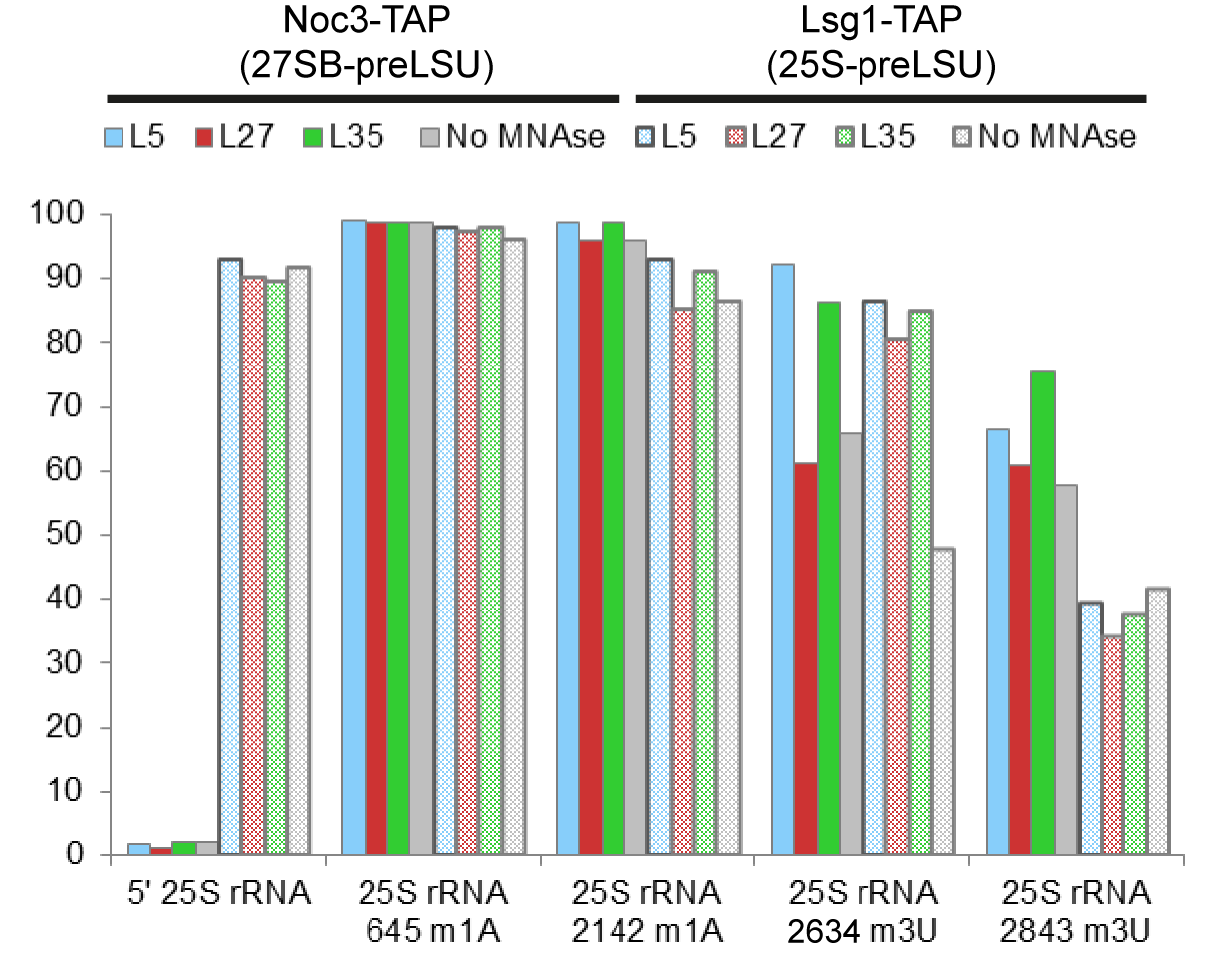

Supplement: S3 Fig — 27SB-preLSUs and 25S-preLSUs were affinity purified via Noc3-TAP or Lsg1-TAP from yeast strains expressing no MNAse (No) or MNAse fused to rpL5 (L5), rpL35 (L35) or rpL27 (L27). After activation of MNAse the positions of generated rRNA 5’ ends were analyzed by random primer extension assays read out by high throughput sequencing. Percent of termination in the indicated rRNA positions (+/- one nucleotide) was estimated as described in Materials and Methods. 3-methyl-uridine is abbreviated with m3U, 1–methyl-adenosine with m1A. (TIF) [file pone.0179405.s003.tif]

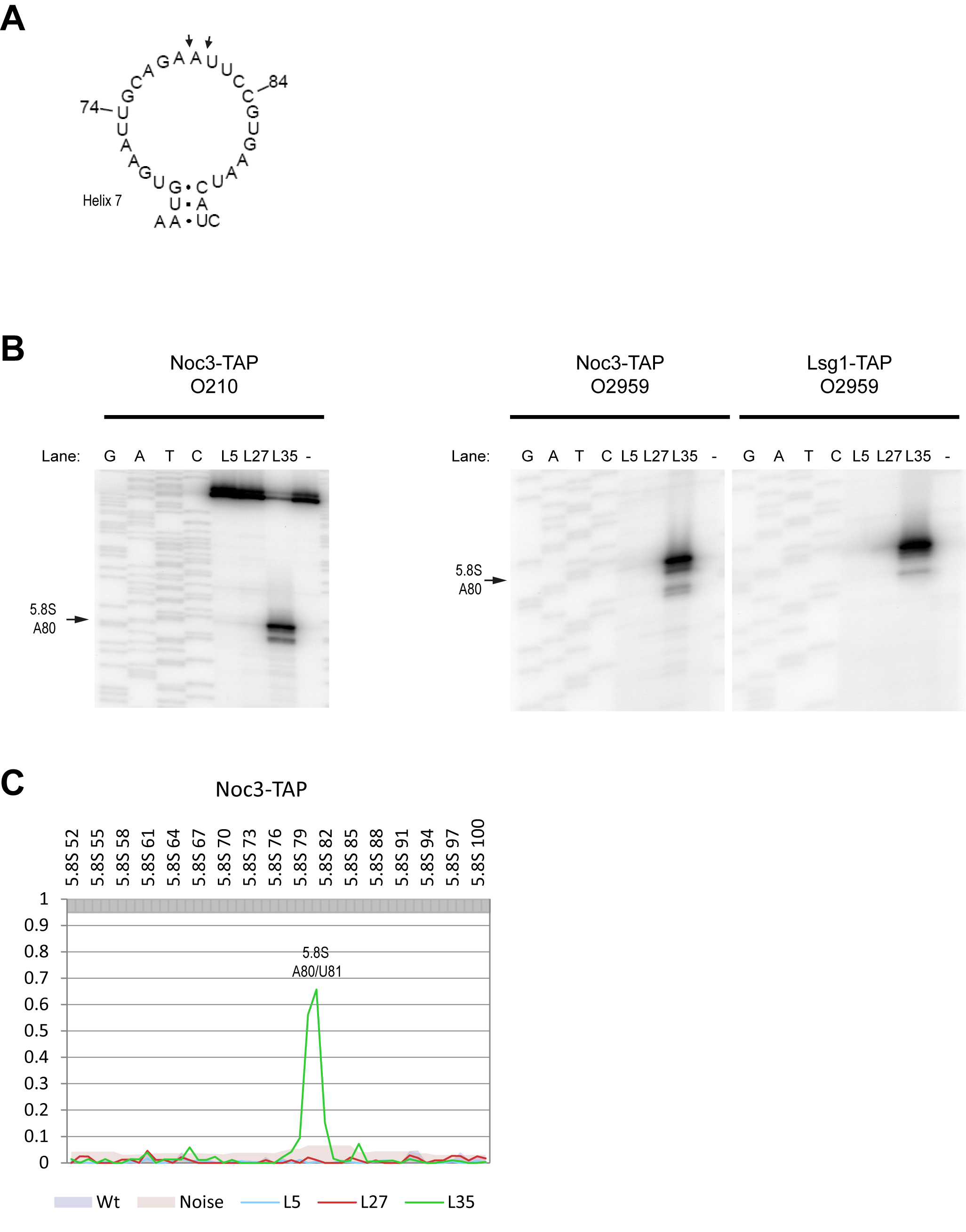

Supplement: S4 Fig — In (A) positions of cleavages are indicated (black arrows) in secondary structure models of the respective rRNA regions. Cleavages previously mapped in helix 38 of 80S ribosomes are indicated by red arrows in S6 Fig. In S4–S11 Figs in (B) results of targeted primer extension reactions using the indicated primers and RNA of 27SB-preLSUs (Noc3-TAP) or 25S-preLSUs (Lsg1-TAP) from strains expressing no MNase (-), or MNase in fusion with rpL5 (L5), rpL27 (L27) or rpL35 (L35) are shown. For each RNA preparation the volume used in the reactions with varying primers was kept constant. Sequencing reactions (lanes G, A, T and C) were performed using the respective primers as described in Materials and Methods. In (C) the termination to coverage ratio determined by random primer extension and high throughput-sequencing (see Material and Methods) is plotted for each nucleotide position in the relevant rRNA region. Nucleotide position 2634, which has a 3-methyluridine base modification, is colored in red at the top of the diagram in S11 Fig. Data were obtained for strains expressing no MNase (Wt), MNase in fusion with rpL5 (L5), rpL27 (L27) or rpL35 (L35). Local noise (Noise) in the high throughput readout of random primer extension reactions was estimated as described in Material and Methods. (TIF) [file pone.0179405.s004.tif]

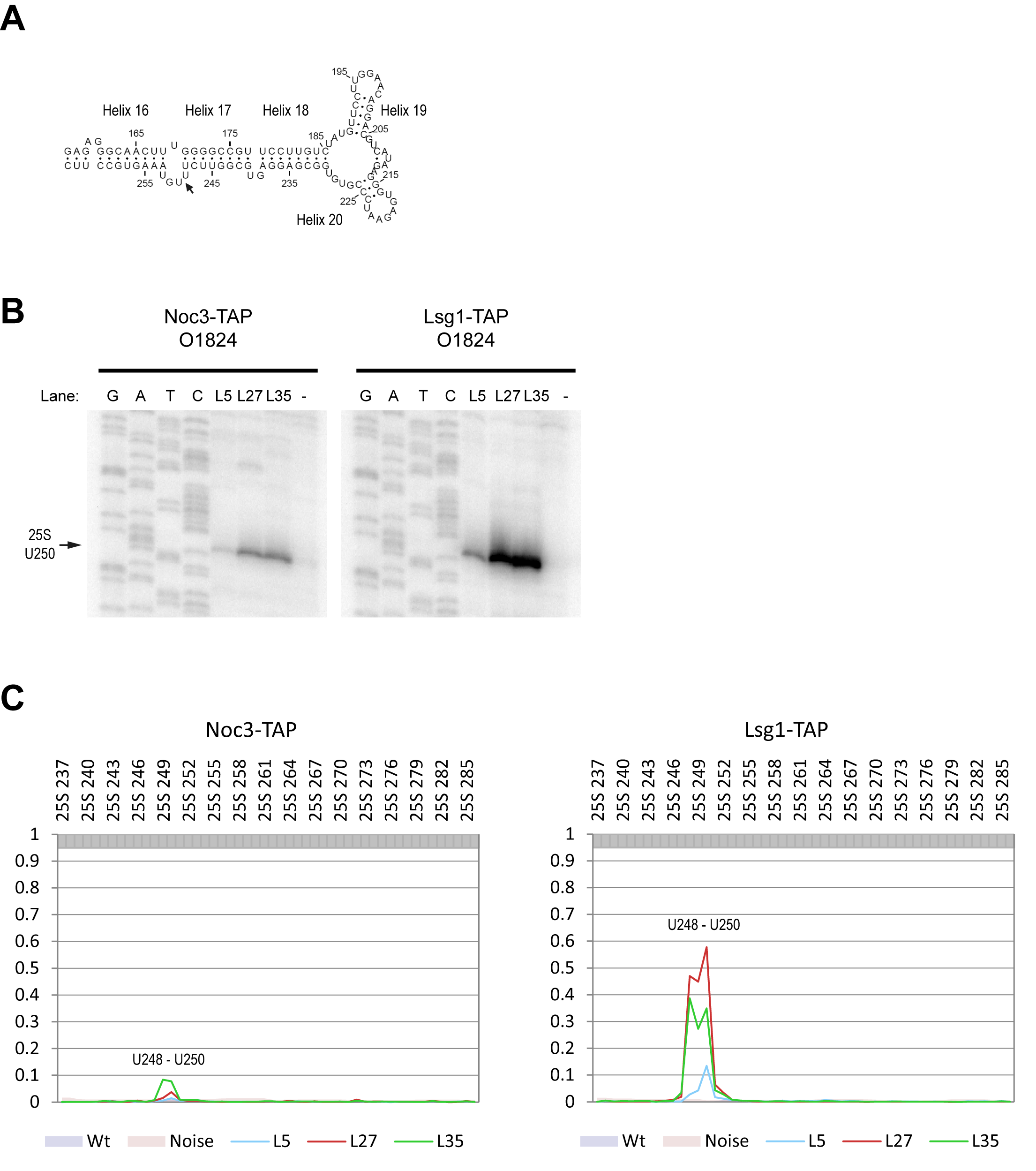

Supplement: S5 Fig — See figure legends for S4 Fig. (TIF) [file pone.0179405.s005.tif]

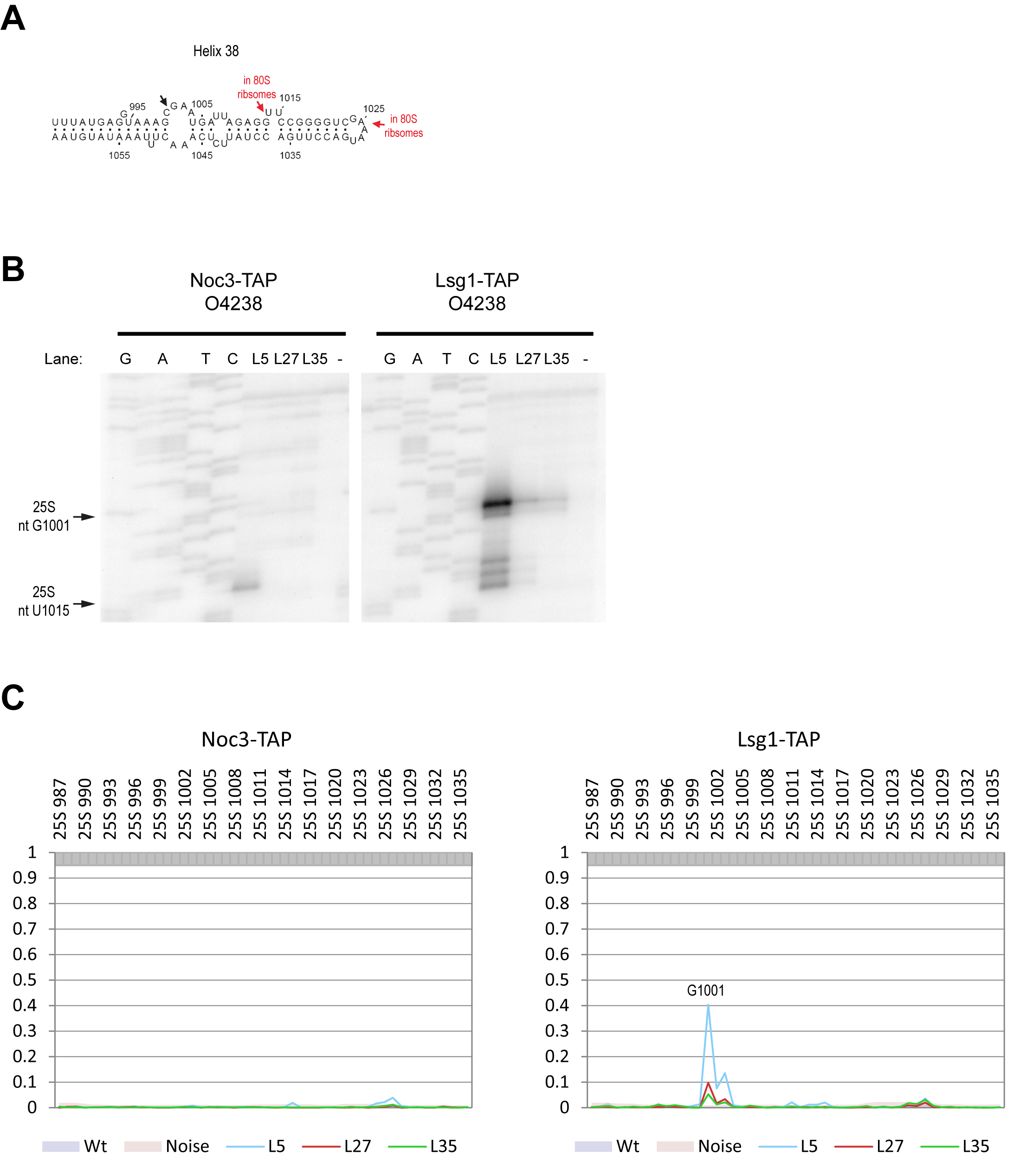

Supplement: S6 Fig — See figure legends for S4 Fig. (TIF) [file pone.0179405.s006.tif]

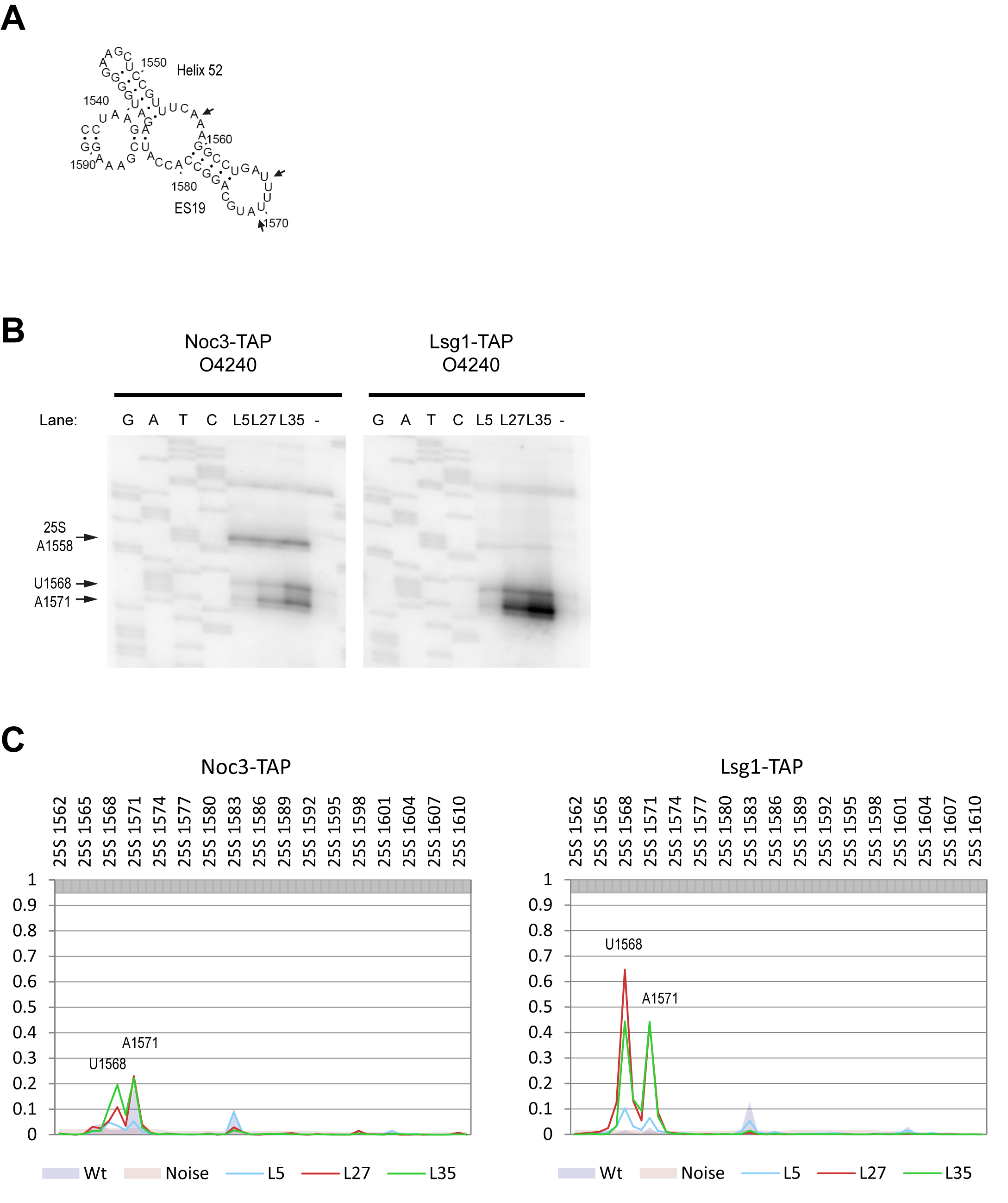

Supplement: S7 Fig — See figure legends for S4 Fig. (TIF) [file pone.0179405.s007.tif]

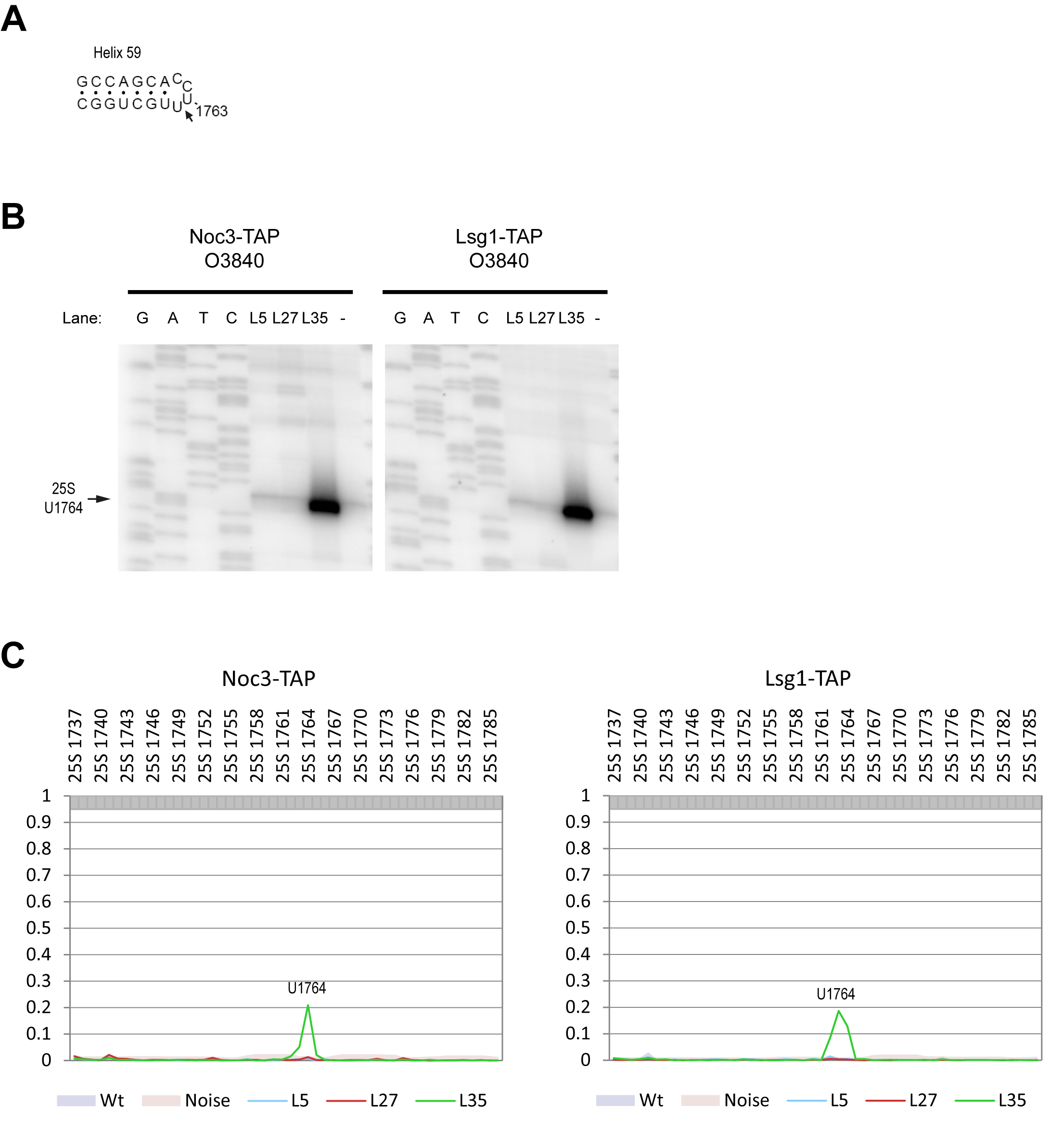

Supplement: S8 Fig — See figure legends for S4 Fig. (TIF) [file pone.0179405.s008.tif]

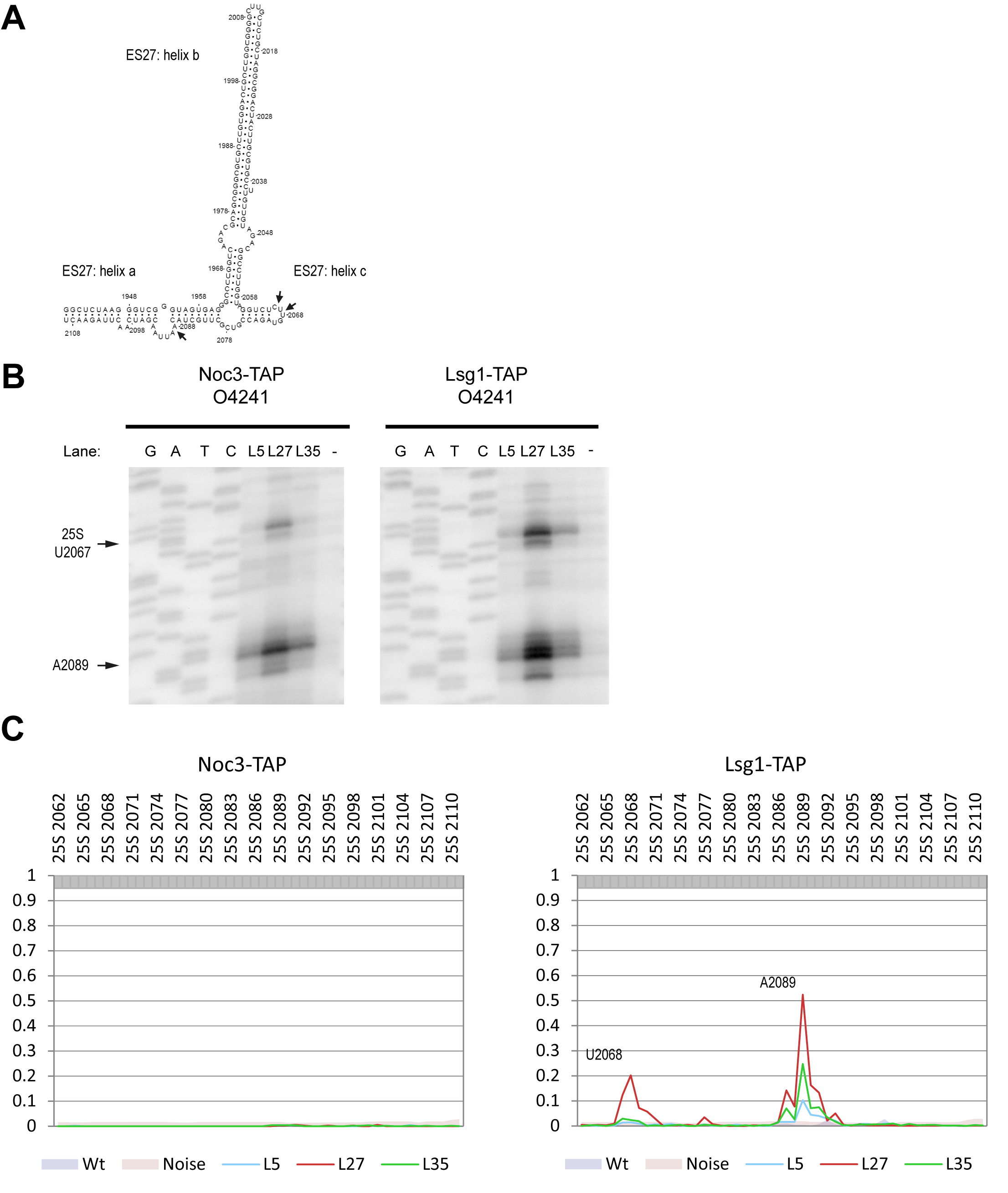

Supplement: S9 Fig — See figure legends for S4 Fig. (TIF) [file pone.0179405.s009.tif]

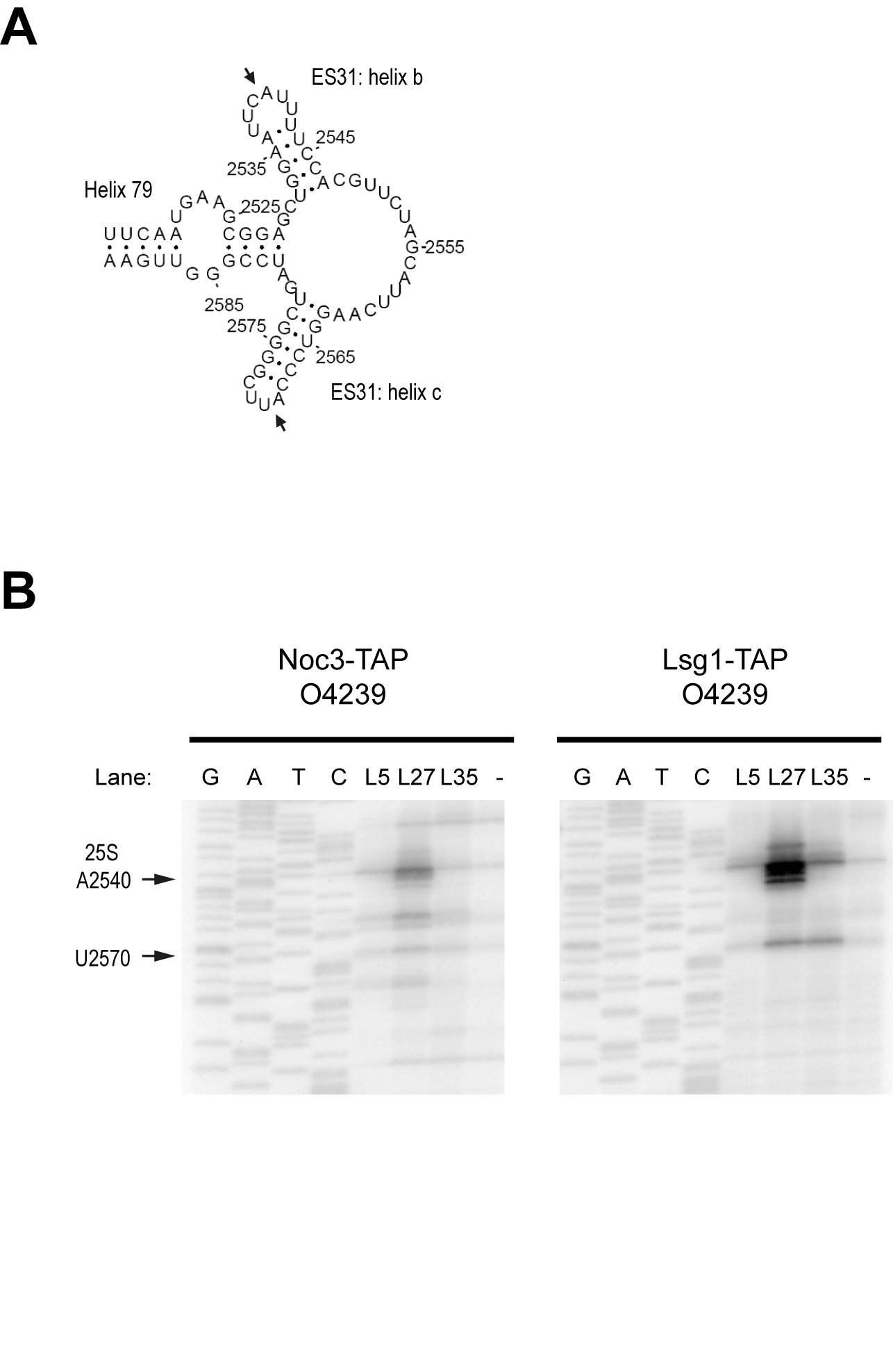

Supplement: S10 Fig — See figure legends for S4 Fig. (TIF) [file pone.0179405.s010.tif]

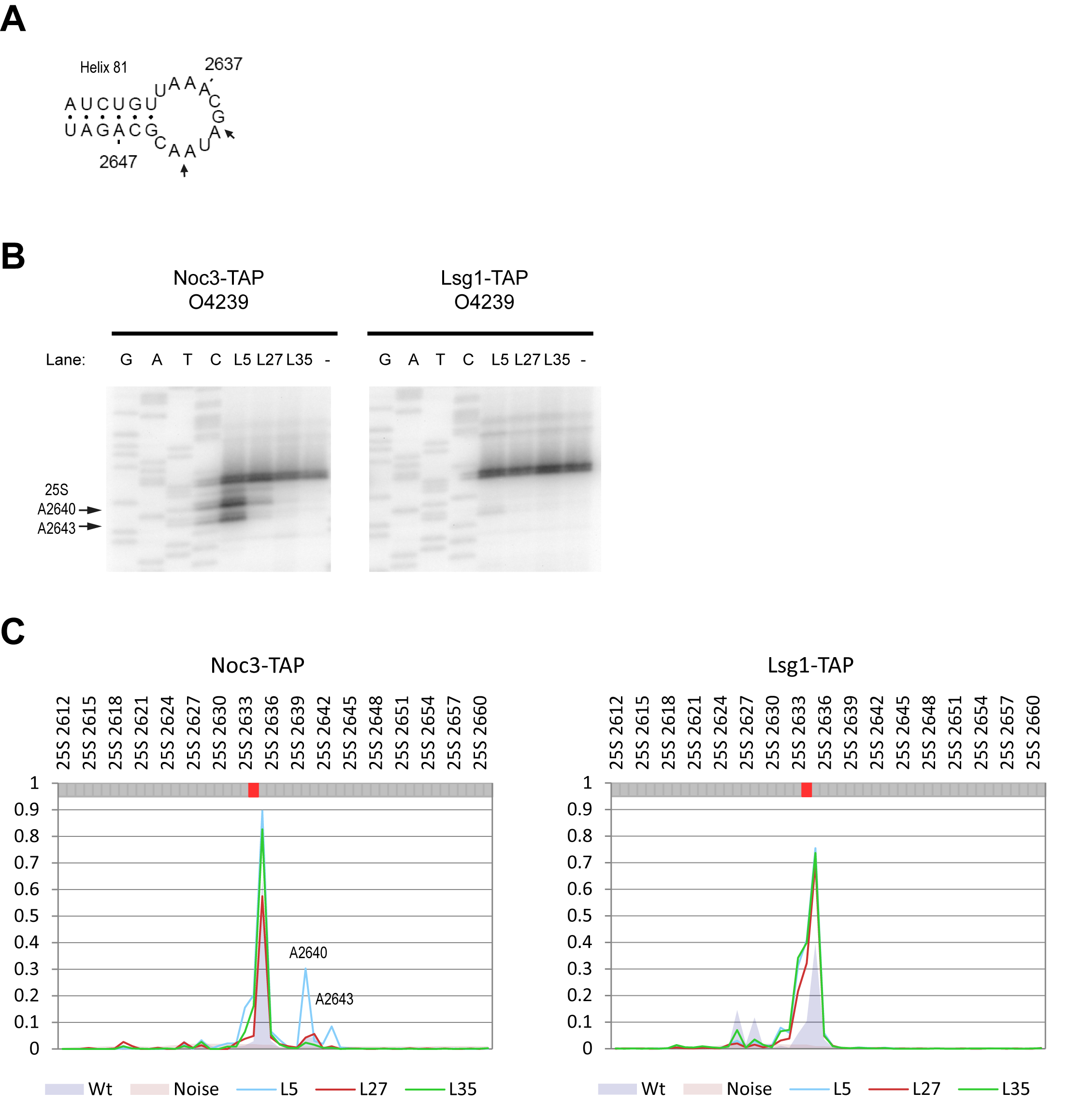

Supplement: S11 Fig — See figure legends for S4 Fig. (TIF) [file pone.0179405.s011.tif]

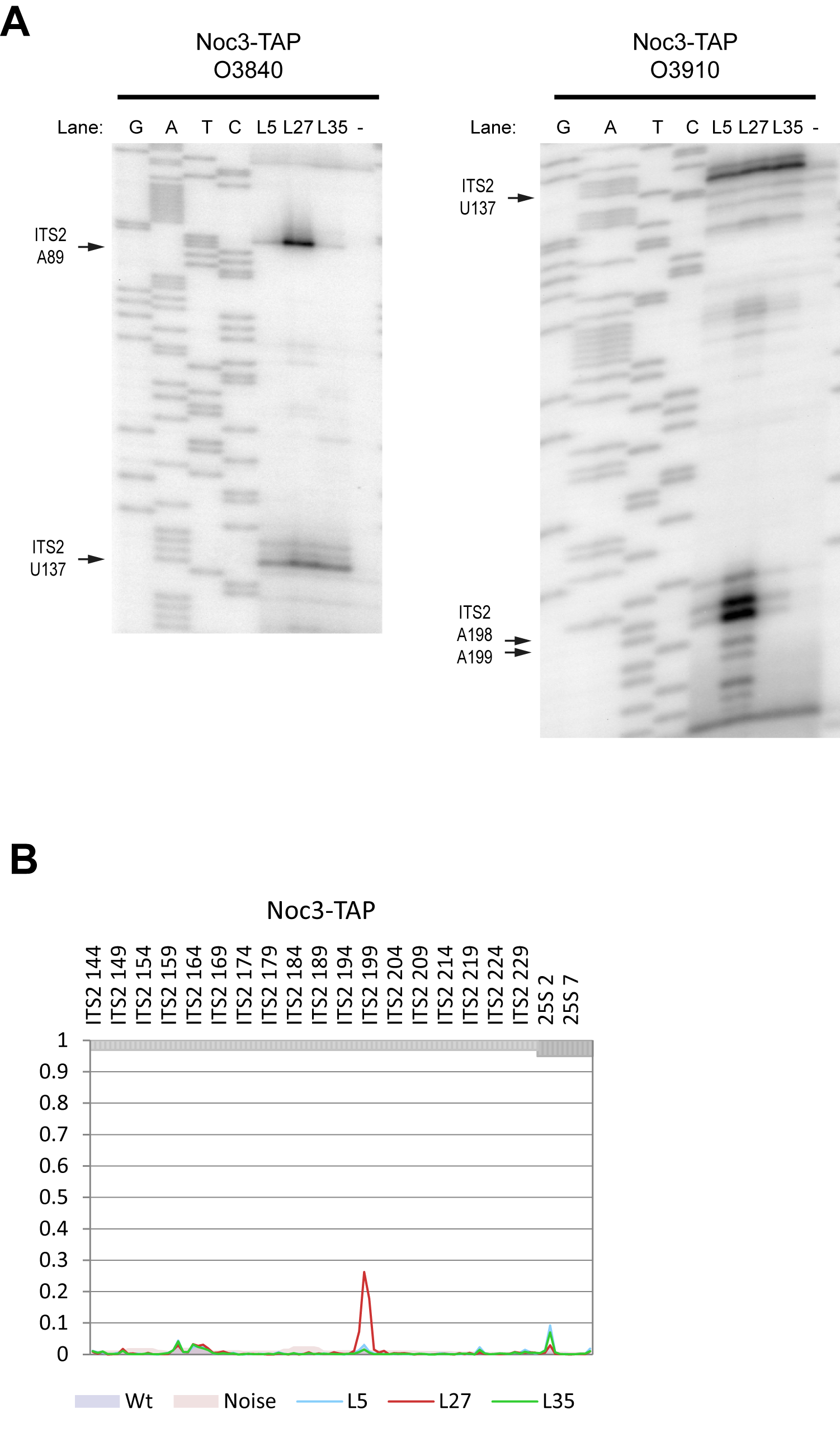

Supplement: S12 Fig — In (A) results of targeted primer extension reactions using the indicated primers and RNA of 27SB-preLSUs (Noc3-TAP) from strains expressing no MNase (-), or MNase in fusion with rpL5 (L5), rpL27 (L27) or rpL35 (L35) are shown. Sequencing reactions (lanes G, A, T and C) were performed with primers O3840 and O3910 as described in Materials and Methods. In (B) the termination to coverage ratio determined by random primer extension and high throughput-sequencing (see Materials and Methods) is plotted for the 3’ region of ITS2. Data were obtained for strains expressing no MNase (Wt), MNase in fusion with rpL5 (L5), rpL27 (L27) or rpL35 (L35). Local noise (Noise) in the high throughput readout of random primer extension reactions was estimated as described in Materials and Methods. (TIF) [file pone.0179405.s012.tif]
